# Concept for the Real-Time Monitoring of Molecular Configurations during Manipulation with a Scanning Probe Microscope

**DOI:** 10.1021/acs.jpcc.3c02072

**Published:** 2023-07-10

**Authors:** Joshua Scheidt, Alexander Diener, Michael Maiworm, Klaus-Robert Müller, Rolf Findeisen, Kurt Driessens, F. Stefan Tautz, Christian Wagner

**Affiliations:** † Peter Grünberg Institut (PGI-3), Forschungszentrum Jülich, 52425 Jülich, Germany; ‡ Jülich Aachen Research Alliance (JARA)-Fundamentals of Future Information Technology, 52425 Jülich, Germany; § Data Science and Knowledge Engineering, Maastricht University, 6229 EN Maastricht, The Netherlands; ∥ Laboratory for Systems Theory and Automatic Control, Otto-von-Guericke-Universität Magdeburg, 39106 Magdeburg, Germany; ⊥ Max Planck Institute for Informatics, 66123 Saarbrücken, Germany; # Machine Learning Group, Technische Universität Berlin, 10587 Berlin, Germany; ▽ Department of Artificial Intelligence, Korea University, Seoul 136-713, South Korea; ° Control and Cyber-Physical Systems Laboratory, Technische Universität Darmstadt, 64289 Darmstadt, Germany

## Abstract

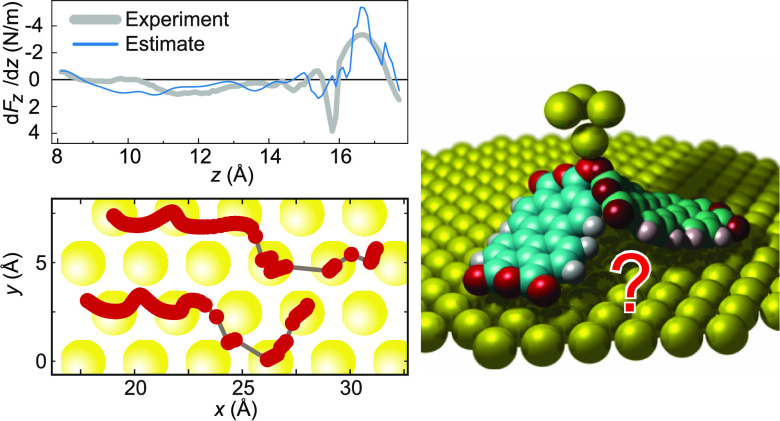

A bold vision in nanofabrication is the assembly of functional molecular structures using a scanning probe microscope (SPM). This approach requires continuous monitoring of the molecular configuration during manipulation. Until now, this has been impossible because the SPM tip cannot simultaneously act as an actuator and an imaging probe. Here, we implement configuration monitoring using experimental data other than images collected during the manipulation process. We model the manipulation as a partially observable Markov decision process (POMDP) and approximate the actual configuration in real time using a particle filter. To achieve this, the models underlying the POMDP are precomputed and organized in the form of a finite-state automaton, allowing the use of complex atomistic simulations. We exemplify the configuration monitoring process and reveal structural motifs behind measured force gradients. The proposed methodology marks an important step toward the piece-by-piece creation of supramolecular structures in a robotic and possibly automated manner.

## Introduction

The ability to handle single molecules as effectively as macroscopic building blocks would enable the fabrication of functional supramolecular structures that are not accessible by self-assembly.^1−4^ A way to approach this goal is the use of a low-temperature scanning probe microscope (SPM) which offers not only imaging but also manipulation capabilities with the tip as the actuator.^5−10^ So far, most SPM manipulation experiments address the rearrangement of atoms or molecules on the surface, typically in a dragging, pushing, or pick–transfer–drop approach.^7,11−15^ The problem of configuration monitoring is not urgent for such experiments since terminal configurations can be imaged, and, moreover, the tip-induced transitions between these configurations occur abruptly, often upon an inelastic excitation of the molecule, which is stochastic in nature. These lateral repositioning methods naturally limit the achievable (supra)molecular structures.

**Figure 1:**
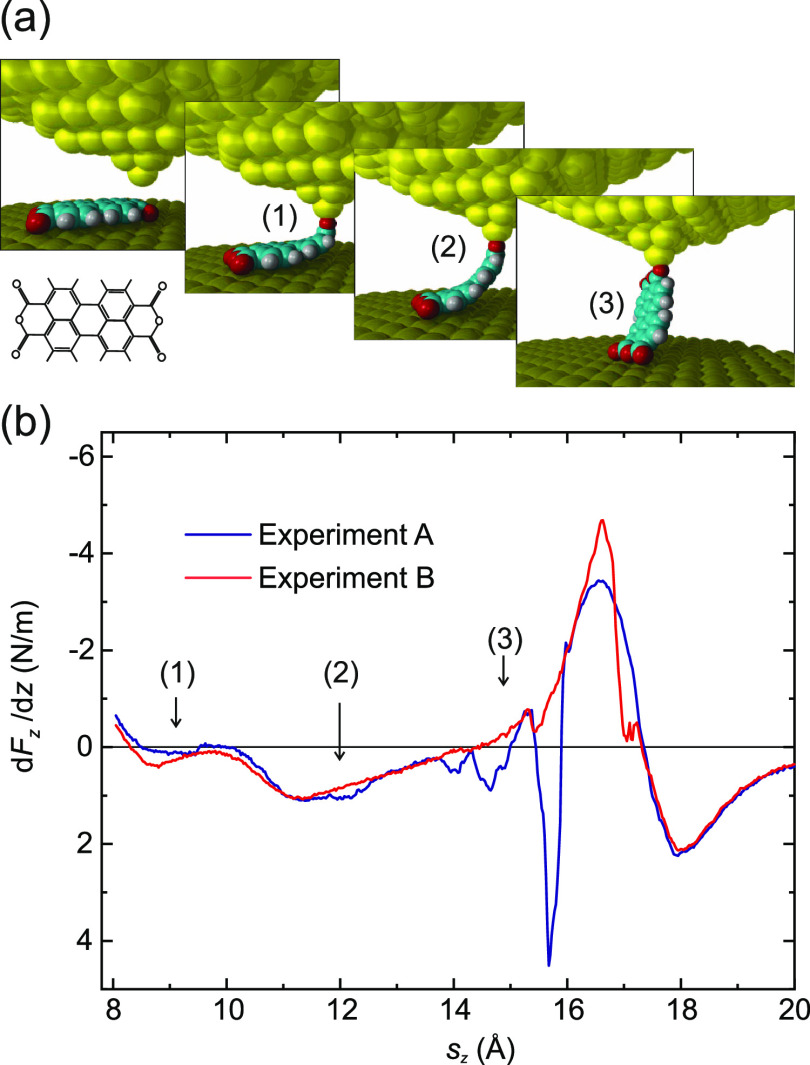
Lifting of a PTCDA molecule. (a) Two-contact manipulation process in which a PTCDA molecule is lifted from a Au(111) surface with the SPM tip. The tip is approached to one of the O_carb_ atoms (left frame), where a chemical bond forms. When retracting the tip on a vertical trajectory (*s*_*z*_ is the *z* coordinate of the tip apex), the molecule is gradually lifted up.^6,16^ (b) Two exemplary experimental d*F*_*z*_/d*z*(*s*_*z*_) curves were recorded for the lifting procedure shown in (a). The three stages of the lifting process (1)–(3) are indicated in both panels. Since PTCDA can take different paths across the surface, the d*F*_*z*_/d*z*(*s*_*z*_) curves differ, particularly in regions where PTCDA is vertical and the O_carb_ atoms come close to the Au(111) surface. The zero of *s*_*z*_ was determined as in ref (17).

To add more degrees of freedom to the accessible configuration space, we have developed the concept of two-contact manipulation in which the tip forms a stable chemical bond with a single particularly reactive atom in a surface-adsorbed molecule (Figure 1a).^1,3,6,10,18^ This “handle” can then be used to apply a force to the molecule and thus manipulate it mechanically (lifting, deforming, translating, detaching) in a manner that is practically deterministic at the experimental temperature of 5 K and allows changing the molecular configuration along continuous trajectories, in contrast to abrupt jumps from one stable configuration to another. Importantly, two-contact manipulation also enables the step into the third dimension away from the surface. While this increases the possible manipulation options considerably,^3,19,20^ the monitoring of the molecular configurations during the manipulation process is very poor. In its dual role as imaging probe and guiding actuator, the SPM tip can only fulfill one task at a time, such that the manipulation happens “blindly”, with raw data from the few measurement channels of the SPM setup (typically force-gradient and conductance data) as the only available information. We have recently shown that even without explicit knowledge of the configuration, certain nanofabrication tasks can be achieved autonomously by using reinforcement learning with sparse feedback.^3^ However, to enable a broader scope of SPM-based robotic nanofabrication, it is indispensable to monitor the molecular configuration during manipulation.

Since imaging is not feasible, configuration monitoring must be based on data sets other than images. These could either originate from the available SPM channels or from additional experimental methods. Any method with single-molecule sensitivity, which is susceptible to the molecular configuration—and does not require scanning the tip—has the potential to provide useful information. Examples include current–voltage, Raman, or optical spectroscopy, as well as force gradient measurements.

How can the link between such data and the molecular configuration be established? On an abstract level, molecular manipulation can be described as a partially observable Markov decision process (POMDP). From this perspective, the tip–molecule–surface junction is a system that transitions from one hidden state, namely, the configuration ***x***, into another by the actions of a decision maker, while the only information about these states can be obtained through observations *u*(***x***), which are the experimental data discussed above. In a POMDP, the acquired information forms the basis for autonomous decision-making aimed at reaching a target state. The policy that links observations and decisions is optimized through obtained rewards.^3^ Configuration monitoring thus provides the information on which rewards can be based or upon which an experimenter can choose actions. It is, hence, the key ingredient for molecular robotic nanofabrication. Here, we present an approach that aims at providing the best possible estimate of the hidden state ***x*** from the sequence of observations *u*_*j*_ made throughout the manipulation process.

The task of configuration monitoring requires combining two strands of information, namely, the manipulation actions taken by the experimenter and the observations made in the simultaneous measurements. To interpret the actions, a state transition model is required, which can predict how the molecular configuration changes upon a certain action. Likewise, to interpret the measured data, an observation model is required that predicts which experimental observation(s) would be made in a given molecular configuration. Both models should, in principle, be statistical in nature since thermal fluctuations could affect state transitions in a statistical way, and experimental noise would affect the observations. Here, we nevertheless use a deterministic state transition model because our experiments were performed at *T* = 5 K. While our observation model is likewise deterministic, we include experimental uncertainty in our solution of the configuration monitoring task.

Because of unavoidable model limitations, experimental noise, and the fact that a molecular configuration is a high-dimensional vector ***x***, while observations might only comprise a single scalar value *u*, a single observation is typically not sufficient to determine ***x*** with high confidence. This challenge can be understood and addressed in the framework of a Bayesian inference approach in which an initial (*prior*) configuration estimate is iteratively refined (*posterior*) as new pairs of actions and observations become available during the manipulation process. We realize this concept using a particle filter (PF) state estimation algorithm.^21^

Three aspects need to be considered when selecting a certain experimental technique or SPM data channel as the basis for configuration monitoring: (1) The method should be capable of producing measurement outcomes on the few-seconds time scale of a typical SPM manipulation experiment, should have single-molecule sensitivity, and a high signal-to-noise level. (2) It is advantageous if the experiment has a large number of possible outcomes (corresponding to different configurations) since this tends to increase the information content
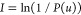
1of an individual measurement, where *P*(*u*) is the probability of measuring *u* (see the section on information gathering). Experiments measuring solely a spin orientation, for example, would be of limited use for configuration monitoring in a large configuration space. (3) Experimental quantities should be preferred for which the observation model can be obtained with limited effort. For a given molecular configuration **x**, the force **F**(**x**) acting on the SPM tip during manipulation is, for example, much easier to calculate than the conductance spectrum I(V) [**x**] or the vibrational spectrum A(ω) [**x**]. This is the reason why we abstain from including molecular junction conductance as a source of information in our approach. Note that we use Roman symbols to refer to the simulated (or estimated) data and italic symbols to denote experimental data.

Here, we describe a configuration monitoring approach in which the observation *u* is the non-contact atomic force microscope (NC-AFM) force gradient *F*′:= d*F*_*z*_/d*z* measured during the manipulation process (Figure 1b). We use a qPlus tuning fork setup^22^ with an oscillation amplitude of 0.3 Å. To avoid irregularities in the configuration space, we perform our experiments in a region of the sample surface that is devoid of defects or step edges and has thus the full symmetry of the Au(111) surface lattice. The PF, which is used to infer the most likely molecular configuration, samples the observation model at a limited number of promising configurations, thus avoiding a search through the entire configuration space . For each sample, the PF compares the actual current observation *F*′ with the predictions F′ of the observation model and refines its state estimate **x**. The deviation between *F*′ and F′, the action of the experimenter, and the state transition model determine which points in configuration space will be sampled in the next PF iteration that is carried out when a new manipulation action changes ***x*** and leads to a new *F*′ value.

To be useful for the control of a manipulation experiment, the PF has to be able to follow the pace with which new action-observation pairs become available, which happens on a time scale of seconds. We achieve this performance by precalculating both the observation and the state transition models for the entire configuration space  and mapping them onto a finite-state automaton (FSA) for quick and structured access. A related important aspect is the choice of the observation and state transition models themselves. If they are based on an atomistic simulation, the computational costs could be very high, rendering a computation of the entire configuration space  unfeasible. If, on the other hand, less complex models are chosen, capturing certain details of the molecular configurations could become impossible. Here, we use a molecular mechanics approach that is atomistic and strikes a balance between accuracy and speed.

We demonstrate the functionality of our configuration monitoring on the example of lifting an isolated PTCDA (3,4,9,10-perylene-tetracarboxylic dianhydride) molecule from a Au(111) surface,^8,16,17^ as shown in Figure 1a. We have extensively studied the manipulation of PTCDA in the past,^1,3,6,8,16−19,23,24^ making it the ideal candidate for this proof-of-concept study. Moreover, we have an optimized force-field simulation for the manipulation of PTCDA on Au(111) at hand, which forms the basis for the observation and state transition models.^8,17,25^ Since this force field is fitted to experimental data, it properly captures the molecule–metal interaction, including many-body contributions.^17^ In this model, the tip is described by its apex atom alone. This is a reasonable simplification since the oxygen atom prefers to bind on top of the tip apex atom and not to a bridge or hollow site.^20^ Moreover, the rest of the tip apex beyond the metal–oxygen bond plays only a minor role in the behavior of the tip–molecule junction.^26^

## Methods

### Primary and Duplicate Configurations

The atomic lattice of the Au(111) surface constitutes the natural frame of reference relative to which all tip–molecule configurations are defined. For simplicity, we neglect the small uniaxial compression of the surface layer which arises from the Au(111) surface reconstruction and assume a strictly hexagonal surface lattice with a periodicity of 2.884 Å, endowed with the respective translational, rotational, and mirror symmetries. This implies that from any given primary tip–molecule configuration **x**, an infinite number of translated, rotated, and mirrored duplicates can be created, all of which are equivalent with respect to the atomic lattice of the surface. If, furthermore, a discrete grid of tip positions **s** is used, the number of primary tip–molecule configurations, which are the ones that need to be computed, becomes finite. This holds under the reasonable assumption of a finite number of relaxed molecular configurations **r** for each **s**, the latter of which is safeguarded by a surface corrugation with distinct minima.

For the discretization of **s** space, we used a hexagonal grid with a step size of 0.120167 Å in the *x*,*y* plane, thus sampling the rhombic Au(111) unit cell at 24 × 24 positions. In the *z* direction, we performed calculations in the interval 5.3 ≤ *s*_*z*_ ≤ 17.7 Å, i.e., the range between a flat-lying and a fully vertical molecule, with a grid step size of 0.1 Å. Altogether, this led to a total of 72,000 allowed tip positions **s**.

### Similarity between Molecular Configurations

When creating the FSA, it is necessary to determine whether a new configuration obtained by relaxation with the molecular mechanics model is already included in the FSA or whether it has been attained for the first time. Two simulated configurations obtained by different manipulation steps will never be precisely the same because the relaxation is terminated by a set of thresholds and, therefore, always incomplete. Since configuration similarity checks are frequently required when the FSA is created, their computational efficiency is important. We use the positions of four atoms for this purpose. Two configurations are considered identical if (1) their tip positions on the discretization grid are the same, (2) the positions of their bottom O_carb_ atoms are the same within an accuracy of 0.2 Å, and (3) the bending direction of the molecule between its anchor points at the tip and in the surface is the same. The third aspect is relevant because the bending is the only remaining degree of freedom of the planar PTCDA molecule once three of its corners are fixed in space. Usually, the molecule would bend toward the surface because of attractive vdW forces, but there are configurations in which PTCDA is bent upwards. They can be reached from the vertical molecular configuration by pushing the tip toward the surface. Subsequently, rotating the bent molecule around the bottom O_carb_–O_carb_ axis toward the direction into which it is not bent preserves the bent.

### Reversibility and Surface Corrugation

When analyzing the aspect of reversibility, it must be remembered that the states and transitions in the FSA depend on the underlying atomistic model. In particular, the model employed for the local Au–O_carb_ interaction has a strong impact on reversibility. Simply speaking, the stronger these bonds are, the more stringent the anchor concept and its consequences are because intermediate states in which the O_carb_ atoms are located between Au atoms (i.e., hollow or bridge sites) will then be suppressed.

In our molecular mechanics approach, we model the Au–O_carb_ interaction for each of the four O_carb_ atoms by a lateral cosine potential^18^
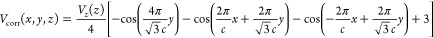
2with *c* = 2.884 Å and a *z*-dependent amplitude *V*_*z*_(*z*). Note that the minima are centered on the Au atoms. The chosen *z* dependence is a simple representation of (weak) chemical bonding; it follows the Fermi-type function *V*_*z*_(*z*) = 40 meV (1 + *e*^(*z* – 2.95Å)/0.22Å^)^−1^. As a consequence, close to the surface, the O_carb_ atoms feel a stronger corrugation. This happens, for example, in an inclined molecular orientation (last panel of Figure 1a).

### Roulette Wheel Selection

We used the so-called roulette wheel selection^27^ for redistributing the particles according to importance weights *W*_*l*_. The algorithm creates a set of *G* intervals, one for each particle **x**_*l*_, with normalized widths *w*_*l*_ = *W*_*l*_/∑_*l*=1_^*G*^*W*_*l*_ that are proportional to the importance weights of each particle. For *l*′ = 1, ··· *G*, the intervals are given by *I*_*l*′_ = [∑_*l*=0_^*l*^′^–1^*w*_*l*_,∑_*l*=0_^*l^′^*^*w*_*l*_], where we defined *w*_0_ ≡ 0. Thus, *I*_1_ = [0,*w*_1_], *I*_2_ = [*w*_1_, *w*_1_ + *w*_2_], *I*_3_ = [*w*_1_ + *w*_2_, *w*_1_ + *w*_2_ + *w*_3_], and so on. Next, a random value between 0 and 1 is assigned to each of the particles, which places them in one of the intervals. Finally, each particle is relocated into the vicinity (up to three neighboring FSA states) of the particle from which the respective interval was created.

### K-Medoid Clustering

The K-medoid clustering technique^28^ is a robust algorithm to cluster points in a metric space. To keep the computation speed high, we do not use as our metric the Cartesian distance in the high-dimensional vector space in which configuration vectors **x** live but define a distance based on fewer quantities which are, in contrast to the components of **x**, largely uncorrelated. Specifically, given two configuration vectors **x**_*A*_ and **x**_*B*_, we use the distance between the tip positions **s**_*A*_ and **s**_*B*_ and the differences between the azimuthal angles of the anchor–tip position vectors ∠_*A*_ and ∠_*B*_ as a measure of the metric distance between **x**_*A*_ and **x**_*B*_,
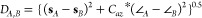
3Note that ∠_*A*_ – ∠_*B*_ is multiplied by a constant *C*_*az*_, which, on average, increases the importance of the azimuthal angles to the same level as **s**_*A*_ – **s**_*B*_.

## Results and Discussion

### Concept

#### Bayes’ Rule

In our proof-of-concept study, configuration monitoring essentially means solving an inverse problem in which the unknown high-dimensional molecular configuration , i.e., the positions of all *n* atoms of the molecule, has to be inferred from the measured *z* component of the NC-AFM force gradient *F*′ (***r***, ***s***) (Figure 1b) which depends on both ***r*** and the SPM tip position  in a way that is described by our observation model. In fact, configuration monitoring has to include the tip position ***s*** into the sought-after quantity  because, in the experiment, ***s*** is typically only known relative to the other tip positions in a trajectory but not relative to the atomic surface lattice that constitutes the fixed reference frame.

In the context of a POMDP, the actions of the experimenter are not based on the knowledge of configuration ***x*** but on their belief about the nature of this configuration. In its Bayesian interpretation, this belief is manifested by a probability distribution over all *N* possible configurations in the configuration space . For a given current experimental observation *u*, the conditional probability that the molecule is in a given configuration **x**_*k*_ is given by Bayes’ rule
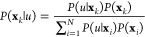
4in which the denominator is simply the probability *P*(*u*). All *P*(u|**x**_*i*_) values, which measure the probability of observing *u* when the molecule is in configuration **x**_*i*_, have to be obtained from the observation model, and *P*(**x**_*k*_) measures the prior probability of **x**_*k*_, that is, the probability prior to observing *u*. If the tip–molecule bond has just been established, the prior could be obtained from an SPM scan recorded before the start of the manipulation. Another example is a prior based on the estimated tip height. If no prior information is available or considered, then *P*(**x**_*k*_) = 1/*N*. Equation 4 expresses a single belief update step in which the probability *P*(**x**_*k*_) is refined from its unconditional prior to its posterior value *P*(**x**_*k*_|*u*).

#### Belief Update along a Trajectory

**Figure 2:**
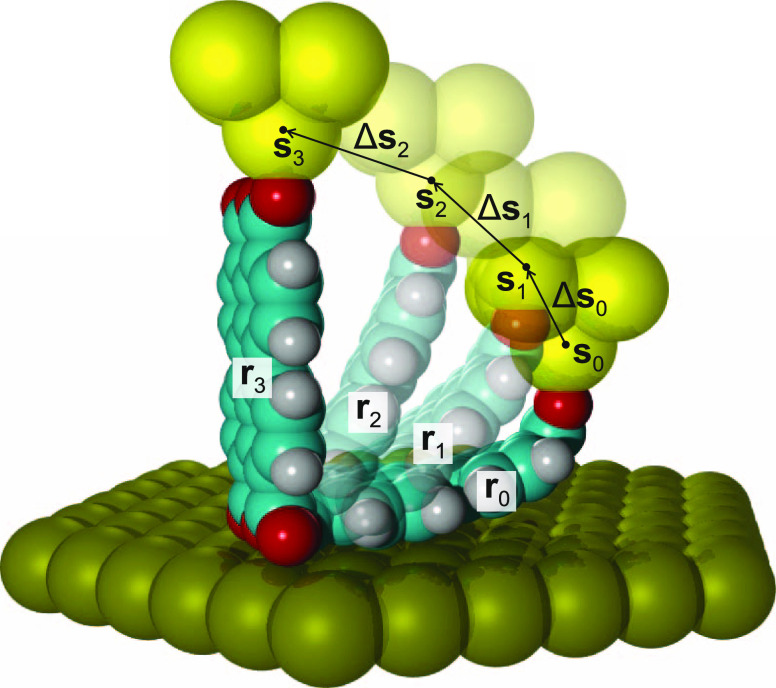
Example of a manipulation trajectory. For this exemplary tip trajectory, the bottom O_carb_ atoms do not move on the surface, such that each of the manipulation steps Δ**s**_0_ to Δ**s**_2_ is reversible in terms of **r**.

Since configuration monitoring means predicting the high-dimensional state, that is, the tip–molecule configuration ***x*** from a low-dimensional observation *u*, it is likely that even substantially different configurations will result in very similar observations. This would lead to a wide probability distribution *P*(**x**_*i*_|*u*) over many **x**_*i*_ (eq 4), which does not favor a single, highly probable configuration. This is unsatisfactory for configuration monitoring. To improve the situation, further belief update steps can be performed based on observations along the entire past trajectory of the manipulation. We represent this trajectory, in accordance with the actual experimental procedure, as a time series of *M* tip translation steps Δ***s***_*j*–1_ for *j* = 1, ··· *M* (i.e., Δ***s***_0_, ··· Δ***s***_*M*–1_) at discrete times *t*_0_, ··· *t*_*M*–1_, carried out between *M* + 1 tip positions ***s***_*j*_ and associated observations *u*_*j*_, both for *j* = 0, ··· *M* (Figure 2). Given such a trajectory, the fully updated belief that **x**_*k*_ is the first state of the trajectory becomes under the Markov assumption
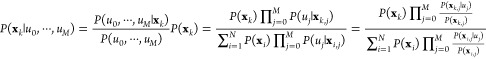
5In the third transformation, we have used Bayes’ rule *P*(u_*j*_|**x**_*k*,*j*_) = *P*(**x**_*k*,*j*_|*u*_*j*_)P(*u*_*j*_)/*P*(**x**_*k*,*j*_). While **x**_*i*_ runs over all configurations in the configuration space , the configurations **x**_*i*,*j*_ are obtained stepwise from **x**_*i*,0_ = **x**_*i*_ and the (deterministic) state transition model *S* as **x**_*i*,*j*+1_ = *S*(**x**_*i*,*j*_, Δ***s***_*j*_), where Δ***s***_*j*_ is the action taken at step *j*. Since the state transition model links all configurations in the numerator of eq 5 into a deterministic sequence {**x**_*k*,0_, ···, **x**_*k*,*M*_}, the probability *P*(**x**_*k*_|*u*_0_, ···, *u*_*M*_) in eq 5 is, in fact, the probability for the entire trajectory. Hence, it is the probability that **x**_*k*,0_ is the first configuration, **x**_*k*,1_ is the second configuration, etc., and **x**_*k*,*M*_ is the current configuration in the trajectory. This is relevant because the current configuration ***x***_*M*_ is naturally the object of interest in configuration monitoring.

#### Computational Complexity

The probability distribution eq 5 for all  represents a complete solution to the configuration monitoring problem. However, this approach will typically fail because it is computationally intractable to evaluate eq 5 for a large configuration space. In particular, the denominator of eq 5 requires *N* × *M* evaluations of the state transition model, where *N* and *M* are on the order of 500,000 and 100, respectively, in the examples discussed below.

Additionally, each evaluation of the state transition model requires the structural relaxation of the molecular configuration, accounting for the new tip position **s**_*j*+1_ ≡ **s**_*j*_ + Δ**s**_*j*_, which is done here via a molecular mechanics model. For the PTCDA molecule with *n* = 38 atoms, this means solving a nonlinear optimization problem in a 114-dimensional space. Although the optimization problem is effectively less complex because the individual atoms’ degrees of freedom are coupled by constraints that follow from the chemical structure of the molecule, performing all optimizations within the few seconds of time *t*_*j*+1_ – *t*_*j*_ elapsing between two steps along the manipulation trajectory is nevertheless unfeasible. This situation calls for an approximation of the probability distribution, which we will describe below.

We take two measures to counter the tremendous computational effort resulting from a brute-force application of eq 5. First, we use a PF to approximate a solution to eq 5, and second, we represent the observation and the state transition models by an FSA, which contains precalculated configurations and corresponding predicted measurement values u from the underlying molecular mechanics model.

#### Particle Filter

Particle filters are one of the methods that can be employed for belief updates in large POMDPs.^29−31^ A prominent application is the localization of mobile robots in a complex environment,^31−33^ based on sensor data and a map as the basis for the observation and state transition models. Two aspects of the PF are central for simplifying the belief update: (1) The probability distribution *P*(**x**_*i*_) over all configurations, which is needed to evaluate eq 5, is never computed in its entirety but instead sampled only for a small subset  of the entire configuration space , in our case *G* = 1500 configurations **x**_*l*_, *l* = 1 ··· *G*, which are called particles. (2) Since even a single evaluation of eq 5 would require a summation across all of , the PF replaces the formally exact calculation of the conditional probabilities *P*(**x**_*l*,*j*_|*u*_*j*_) in eq 5 by a relative measure of probability which is based on the likeness between predicted and measured observations F_*l*,*j*_^′^ and *F*_*j*_^′^ for all **x**_*l*,*j*_, where F_*l*,*j*_^′^ is obtained from the (deterministic) observation model *U* as F_*l*,*j*_^′^ = *U*(**x**_*l*,*j*_). Here, relative probability simply means that a sampled configuration **x**_*l*,*j*_ with a high correspondence between *F*_*j*_^′^ and F_*l*,*j*_^′^ has a higher probability of resembling the unknown configuration ***x***_*j*_ than a configuration **x**_*l*′,*j*_ with a low correspondence. The calculation of this correspondence requires the definition of a metric over the space of all observations.

The aspect that the prior probability of step *j* + 1 is the posterior probability of step *j*, which is implicitly contained in the considerations that lead from eqs 4 to 5, is contained in the PF via a weighted distribution of sampling points **x**_*l*_ at which the prior is evaluated. Regions in , which have a higher prior probability, are sampled more densely. In this way, one only obtains a probability distribution over likely configurations and not across the entirety of , but this is no principal shortcoming if the task is to find the best representation of the actual molecular configuration ***x***_*j*_ in the experiment. However, since the sampling steps of the PF involve some randomness, the PF does not necessarily find the globally best estimate of ***x***_*j*_ but only a good approximation. Below, we will exemplify this aspect using experimental data.

#### Finite-State Automaton

To follow a given tip trajectory, the PF needs information on how the molecular configurations **x**_*l*,*j*_ transform into each other upon any single tip displacement step Δ**s**. This information is provided by the state transition model. While our molecular mechanics model could directly serve as the state transition model, it would be too slow for configuration monitoring, as detailed above. We circumvent this problem by precomputing all possible transitions in the entire configuration space. To manage this data set, we insert a layer of abstraction between the atomistic simulation and the PF, namely, an FSA. After discretizing the state space, the transition model can be represented by a directed graph in which edges equal transitions and nodes equal states. Since our model is deterministic, the graph is rather sparse with, at each node, only one edge for each possible tip translation step Δ**s**. In a probabilistic model, on the other hand, the graph would be edge-weighted and much denser because a tip step could induce a transition to several different states **x**_*k*_ with transition probabilities *P*(**x**_*k*_) > 0. Given these properties, the task of generating and storing the transition model can be performed by an FSA.^34^ Since the calculation of the force gradients F′(**x**_*k*_) for each configuration **x**_*k*_ is a byproduct of the molecular mechanics relaxation procedure, the observation model is generated and stored alongside the transition model by the FSA.

#### Information Gathering

As we have discussed above, a single action-observation pair is typically not sufficient to determine a given configuration ***x***_*k*_ with high certainty, such that the entire trajectory needs to be taken into account. This poses a problem in situations where no such trajectory is available (yet), although a decision about the next steps of the manipulation process nevertheless requires knowledge of ***x***_*k*_. Hence, the question arises whether a sufficiently long trajectory can be generated by manipulation with the sole purpose of information gathering before returning to the initial (and previously unknown) configuration ***x***_*k*_ at the end.

Information-gathering policies are a recurring topic in POMDPs.^35,36^ Given the FSA, one could, in principle, compute the optimal information-gathering trajectory, in which the choice of each next step Δ***s*** would be based on the sequence of *F*′ values measured so far. However, computing this strategy would lead to a combinatorial explosion since there exist 8^*l*^ possible trajectories of length *l* for a single starting configuration alone. Moreover, the resulting strategy would only be valid for the specific FSA it has been developed for and not transferable. Therefore, we take a more general approach here and calculate the number of steps *K* that would, on average, be required to identify a molecular configuration unambiguously, resorting to our specific FSA only for parameterization. The knowledge of *K*, together with the aspect of reversibility discussed below, can then form the basis for the design of appropriate information-gathering trajectories.

Following the concepts pioneered by Shannon,^37^ we equate the information necessary for unambiguous identification of the configuration with the information content of force gradient measurements along a sufficiently long trajectory (on average *K* steps). Modeling the configuration as a discrete random variable *C* for which the uniform probability of each of the *N* configurations is *P*_*C*_ = 1/*N*, the information content in natural units (nats) of knowing the configuration is *I*(*C*) = ln(1/*P*_*C*_) = ln(*N*) (cf. eq 1). The measurement process, on the other hand, involves continuous distributions of a measured quantity *Y* (here *F*′) and the inherent noise *Z*. The true signal *X* and the noise *Z* add up to the measured signal, *Y* ≡ *X* + *Z*. To calculate *K*, we need to determine the mutual information *I*(*X*, *Y*) of *X* and *Y*, that is, how much is learned about *X* from knowing *Y*.^38^ Using the concept of differential entropy to analyze the information content in continuous distributions, we obtain the simple relation *I*(*X*, *Y*) = ln(σ_*Y*_/σ_*Z*_),^38^ which depends only on the standard deviations of *Y* and *Z* (see Appendix).

If this information is obtained at each step of the information-gathering trajectory, we simply get *K* = *I*(*C*)/*I*(*X*, *Y*). However, in practice, more steps will be required since the true values, *X*_1_, *X*_2_, ···, at subsequent steps are typically correlated. As a consequence, the additional information gained from every but the first measurement is less than *I*(*X*_1_, *Y*_1_) because, for all *i* > 1, some information about *X*_*i*_, specifically *I*(*X*_*i*_, *Y*_*i*–1_), was already known from the *Y*_*i*–1_ measured at the previous step. Since for all *i* > 1 only the reduced information Δ*I*(*X*_*i*_, *Y*_*i*_) = *I*(*X*_*i*_, *Y*_*i*_) – *I*(*X*_*i*_, *Y*_*i*–1_) is obtained from measuring *Y*_*i*_, a larger number of steps is needed to obtain the information *I*(*C*) required for unambiguous identification. In the Appendix, we derive the relation
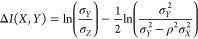
6which depends on the standard deviations of the involved distributions and on the Pearson correlation coefficient ρ between subsequent *X*_*i*_ along the trajectory. Knowing Δ*I*(*X*, *Y*), the required length of an information-gathering trajectory can be computed as
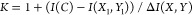
7since the first measurement yields the full information *I*(*X*_1_, *Y*_1_), while all subsequent measurements only yield the difference Δ*I*. As we will later show in the section on information gathering, values of up to *K* = 60 are possible in our experiments, raising the question of whether an information-gathering trajectory of such a length can be successfully reversed, thus safely returning to the initial configuration ***x***_*k*_, for the determination of which the trajectory was performed in the first place.

#### Reversibility

**Figure 3:**
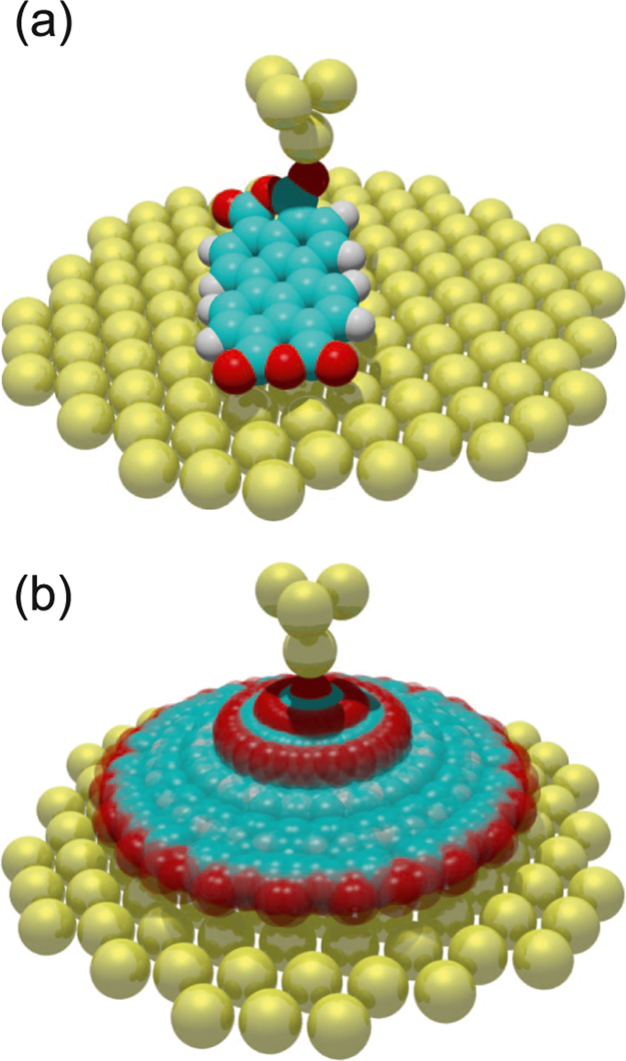
Atomistic simulation. (a) Visualization of a single stable molecular configuration at *s*_*z*_ = 10 Å. (b) Visualization of a subset of , which contains all 47 stable molecular configurations resulting from our molecular mechanics state transition model for a given exemplary SPM tip position at *s*_*z*_ = 10 Å. The primary degree of freedom is the azimuthal angle of the molecule.

The large number of stable molecular configurations at a single tip position in Figure 3b illustrates that returning the tip to its original position, ***s***_*k*_, alone does not guarantee that ***r*** and, thus, ***x*** return to their initial values as well. On the contrary, after a sufficiently long random manipulation trajectory, the probability of returning to ***r***_*k*_ is rather small, as is shown in the Supporting Information. On the other hand, intuition tells us that a sufficiently small tip translation step Δ***s*** should usually be reversible with respect to ***r***. If all individual steps Δ***s***_*j*–1_ = ***s***_*j*_ – ***s***_*j*–1_ of a manipulation trajectory were reversible in such a way, the molecule could be returned to its initial state by reversing the entire trajectory step by step. We will address this aspect in the Supporting Information.

To answer the question of which configurations and which trajectories allow reversibility in practice, we also have to consider some specific properties of the molecule–surface system for which the manipulation is performed. Two-contact manipulation of PTCDA proceeds by approaching the tip to one of the carboxylic oxygen atoms (O_carb_) of the molecule, whereupon a tip–O_carb_ bond forms spontaneously due to the reactivities of the oxygen atom and the undercoordinated Au tip apex atom (Figure 1a). O_carb_ is also expected to form bonds with the Au atoms in the close-packed Au(111) surface if the Au–O_carb_ distance is not too large. While these bonds will be weaker due to the higher coordination of the Au atoms in the surface,^26^ they still have a considerable impact on the measured force gradients (Figure 1b). Such molecule–surface bonding is more prominently observed on the Ag(111) surface, where it leads to a distortion of PTCDA, with the O_carb_ atoms bending toward the surface to a distance of 2.66 Å above the topmost atomic plane of the substrate.^39^ In its adsorbed state on Au(111), the O_carb_ atoms of PTCDA are, on the other hand, found at a larger distance from the surface,^39^ such that we expect considerable local bonding to appear only once the O_carb_ atoms approach the surface when the PTCDA molecule is inclined in the lifting process (right panel in Figure 1a).

Based on this assumption of a chemical molecule–surface interaction via O_carb_ atoms, we can postulate a simple model of reversible manipulation. We assume that any manipulation step Δ***s***_*j*_ is reversible in terms of ***r*** if the bonds between the two bottom O_carb_ atoms (the ones at the side of PTCDA to which the tip is not attached) and two Au atoms in the surface stay intact. An example of such reversible steps is shown in Figure 2. If, however, an O_carb_ atom changes its binding partner to another Au surface atom as a result of a tip translation Δ***s***_*j*_, the respective change from ***r***_*j*_ to ***r***_*j*+1_ is assumed to be ratchet-like and discontinuous and thus irreversible (because of being hysteretic) upon returning the tip to ***s***_*j*_. We will test the validity of this assumption when discussing the fundamental properties of the entire state space  of all simulated tip–molecule configurations. For a set of exemplary trajectories designed for information gathering, we moreover will determine the probability of returning to the starting configuration **x**_*j*=0_ upon trajectory reversal (Supporting Information). This requires an analysis of the generic structure of the directed graph of the FSA.

### Finite-State Automaton

#### Motivation and Objective

Coming back to the issue of computational complexity in configuration monitoring, we analyze the situation in more detail. The fastest atomistic simulations available are force-field methods. They are based on rapidly computable analytical potential energy functions and are often used in molecular dynamics (MD) simulations. Recent advances in hardware and broadband communication have enabled new applications of such MD simulations: animations based on precomputed MD trajectories can be streamed online,^40^ and virtual reality environments have been combined with interactive MD to enable intuitive, user-controlled ad hoc simulations.^41^ In the latter case, the equations of motion have to be solved fast enough to allow all atoms to follow the user input adiabatically. At first sight, this is similar to the requirements of configuration monitoring. However, the PF applied in configuration monitoring would require running hundreds of force-field simulations in parallel (one for each particle) with the external input of a constraint, for example, the SPM tip position, changing at a rate similar to the user input in interactive MD. It is currently unfeasible to realize this computational effort in a lab environment. This holds even when applying the substantial simplification of zero temperature, which turns MD into pure structural optimization, as done in this work. The task would become utterly impossible if a more sophisticated ab-initio method were chosen to map out the space  of possible molecular geometries and calculate the force gradients F′. For each relaxed tip–molecule configuration, the respective density functional theory calculation would take minutes to hours on a supercomputer. We note that machine-learned force fields (e.g., ref (42)) may be an alternative along these lines for future studies. As a radical solution to this dilemma, we therefore completely detach the simulations from the PF state prediction and instead precalculate and store simulation data.

#### Reachable Configurations and Anchor Sets

We continue with some initial thoughts on the FSA. Since it is not known a priori at which points the PF will sample the state space , it is required that all reachable tip–molecule configurations in  are computed in advance and stored in a rapidly accessible form. Here, reachable denotes all configurations that can be accessed from a given starting configuration **x**_0_ in which the molecule is flat on the surface via any possible sequence of tip translation steps, Δ**s**_0_, Δ**s**_1_, ···, and subsequent structure relaxations. Relaxation is required at each step because a change in **s** changes the potential energy landscape of the entire tip–molecule–surface systems.

Two notable facts about reachable configurations should be stressed. First, it cannot be excluded that there are stable configurations that cannot be reached starting from a molecule that is flat on the surface. Since, however, all manipulation experiments considered here start by contacting a flat molecule, we assume that configurations that cannot be reached by our discovery procedure will also not be obtained in an experiment. Second, reaching a configuration in the described way is not trivial. For example, the chirality of the tip–molecule–surface system, which arises since the O_carb_ atom bound to the tip is located on neither of the two mirror axes of PTCDA, can only be switched by rotating the molecule around the bottom O_carb_–O_carb_ axis onto its other side via a configuration in which the molecule is vertical on the surface.

The criterion of being reachable defines an infinite continuum of configurations. To cope with this problem, we discretize the space of tip positions **s** and take advantage of the symmetry and periodicity of the Au(111) surface lattice by defining a finite set of primary configurations and, correspondingly, an infinite set of symmetry-equivalent duplicates (see Methods). Since  can thus be divided into subsets, each containing infinitely many symmetry-equivalent configurations, it is possible to freely select one member of each subset as its primary configuration. These primary configurations form a subset  with the property that no two elements of  can be transformed into each other by a symmetry operation.

**Figure 4:**
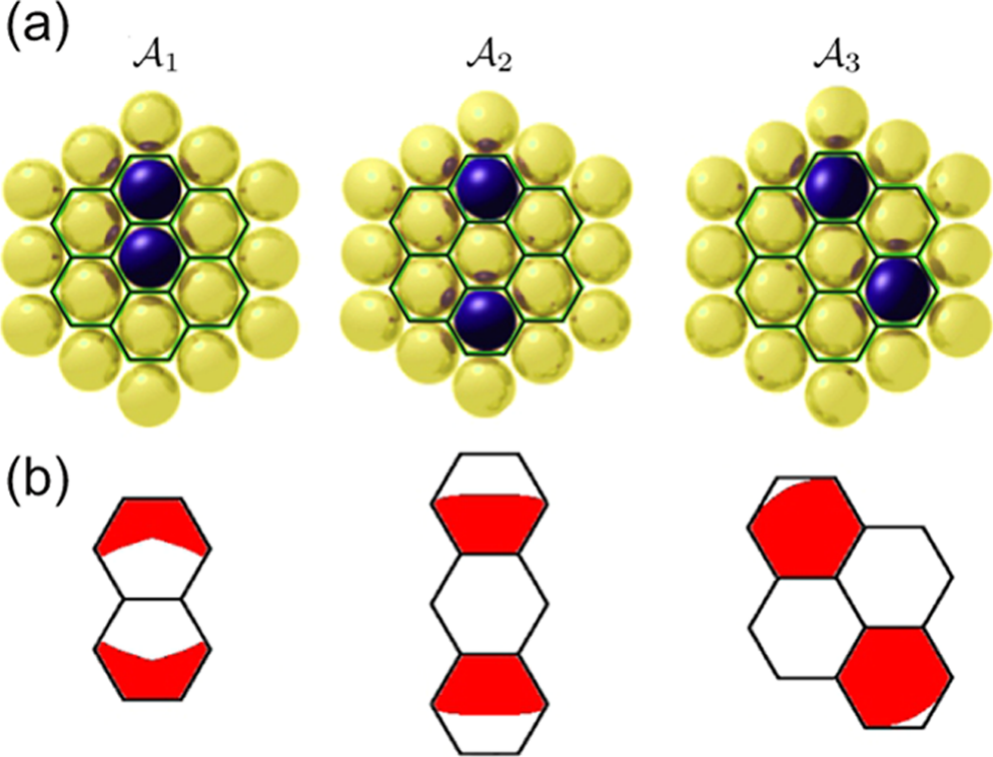
Anchor configurations. (a) The three unique anchors found for PTCDA on Au(111), including their respective Voronoi diagrams. (b) The reachable areas for the three anchors. Red indicates possible lateral positions of the two O_carb_ atoms when PTCDA is bound to the respective anchor (compare Figure 6b). These reachable positions are primarily defined by the intrinsic separation of the two O_carb_ atoms (4.5 Å).

Given the freedom to configure , we opted for building the notion of reversibility directly into . For that purpose, we choose  such that transitions between its elements should, whenever possible, be reversible according to the model of Au–O_carb_ bonds developed above (Figure 2). Hence, we form  by including all configurations in which the two bottom O_carb_ atoms of PTCDA bind to a specific pair of Au atoms in the surface; the latter we refer to as the anchor. Since there will be molecular configurations for which such bonds are not well defined or absent, we stipulate that an O_carb_ atom is always considered as “bound” to the Au atom in the 2D Voronoi cell^43^ (Wigner-Seitz cell) in which it is located (Figure 4). Thereby, the vertical Au–O_carb_ distance is not taken into account. Using this convention, we identify in the simulation three nonequivalent anchors that PTCDA can bind to (Figure 4a). Consequently,  consists of three anchor sets, . With this definition of , we have specified which states should be included in the FSA. The final step is, therefore, the construction of the FSA itself. Note that the anchors do not have to be specified a priori but are discovered during the creation of the FSA. In practical terms, the anchor also provides a reference frame for the definition of the relative *xy* coordinates of **s** and **r**, which simplifies transformations among symmetry-equivalent configurations.

#### Implementation of the FSA

As discussed above, the states  and the transitions between these states form a directed graph, which in turn can be built up by the FSA.^34^ An FSA is a mathematical model of computation that operates on a sequence of input symbols. This input determines a transition from one state of the automaton to another. In our case, tip–molecule configurations **x** are the states of the FSA, and tip steps to neighboring points on the grid of discrete tip positions represent the transitions ***τ*** ≡ (**x**, x*), with **s** stepping to s* and **r** changing to r*. Using the original notation of finite-state transducers by Roche and Schabes,^34^ we can describe our FSA as *M* ≡ (Σ_1_, Σ_2_, , **x**_1_, ) where the input alphabet Σ_1_ = {*a*_1_, ···, *a*_8_} contains all possible actions, i.e., all tip translations *a*_*d*_, where *d* is one of the eight directions along which the SPM tip can move on the hexagonal grid (see Methods).

Σ_2_ is the output alphabet. In our case, we return the complete state **x** for the updated tip position together with the simulated F′ = *U*(**x**) value.

 is the set of states consisting of the primary configurations as defined in the previous section. Initially, the size of this set is unknown because  will be gradually filled up during the building of the FSA.

 is the initial state, which, in our case, is an arbitrarily chosen configuration in which the molecule rests flat on the surface.

 is the set of final states. In our case, there are two types of final states. The first type is encountered when the tip position **s** moves out of the allowed range of *z* coordinates. The second type of final state represents those states x* that belong to a different anchor set from that of **x**.

 is the set of transitions
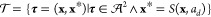
8from a state **x** to a neighboring state x*, where an input from Σ_1_ is used in the state transition model *S*:**x** × Σ_1_ → x*. Note that it is impossible to have a transition ***τ*** = (**x**, **x**) after applying an input *a*_*d*_.

**Figure 5:**
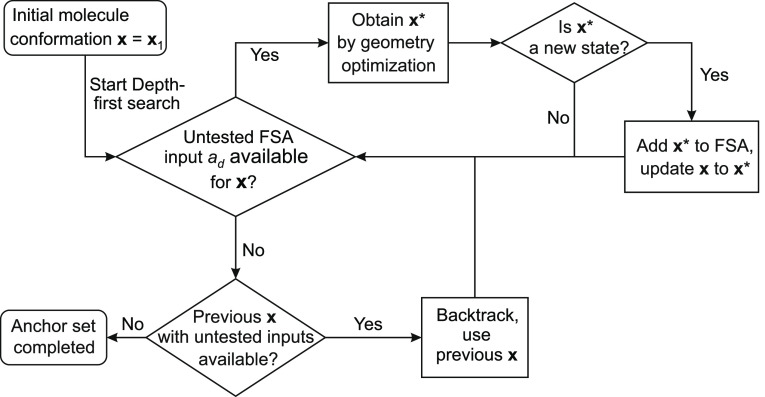
Flowchart of the algorithm that creates the FSA and its underlying graph. This simplified scheme shows the creation of a single anchor set . Once the depth-first search is complete, the algorithm will repeat with a new initial state **x**_1_ from the next anchor set.

The algorithm builds the FSA and its underlying graph by sequentially applying all tip translation steps in a depth-first search^44^ (Figure 5), starting from state **x**_1_. Applying an input *a*_*d*_, the tip moves from **s** to s*. At s*, the respective molecular configuration r* is computed by geometry optimization with our molecular mechanics model, starting from the previous configuration **r**. The resulting tip–molecule configuration x* found in this way is added to the FSA (or the graph, respectively) as a new state. All eight tip translation steps *a*_*d*_ are applied sequentially in this manner. Since there are several **r** and several inputs *a*_*d*_ for any given **s**, each point **s** on the grid of tip positions must be visited multiple times. If a state is encountered which has been visited before (see Methods) and to which all possible inputs have already been applied the algorithm backtracks, that is, it continues with the last visited state that still has untested inputs. When all inputs to all states have been tested, the algorithm stops since the FSA is completed.

A final state  normally stops the algorithm, but in our case, it only stops the current branch of the depth-first search through state space, resulting in backtracking to a new branch or, ultimately, in the completion of the FSA and its underlying graph. Whenever a final state of the second type, i.e., a state in a different anchor set, is encountered, the algorithm not only backtracks but also stores the new state x* to a separate branch of the depth-first search at the root level. Only after the current anchor set _*q*_ has been completed, the algorithm continues with the states x* that generate new anchor sets, thus filling all anchor sets  sequentially. For these states x*, the FSA also stores additional information on the translation and rotation from the anchor set  to the anchor set , as this is required to transform the tip position **s** relative to the old anchor to the tip position s* relative to the new anchor. Hence, the second type of final states yields information on how and where in **s** space the various anchor sets are connected and which transformations switch between them.

**Figure 6:**
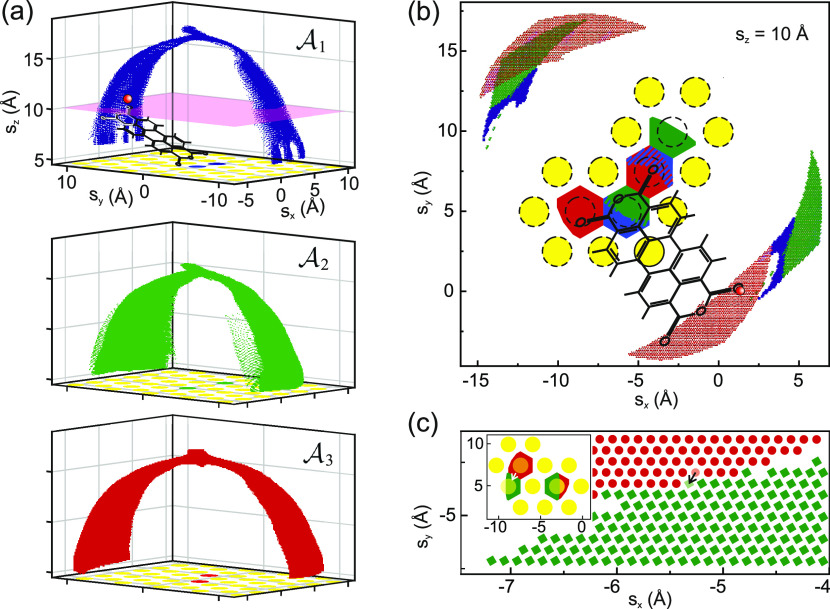
Anchor sets. (a) Graphic representation of all tip positions **s** in the three primary anchor sets . The lateral positions of Au atoms are displayed in yellow, with the anchor atoms in the corresponding color. As exemplified in the upper panel, each point in the displayed cloud of tip positions stands for a full configuration **x** of the tip–molecule–surface junction. (b) Horizontal cut through the **s** cloud of the three anchor sets at s_*z*_ = 10 Å (the pink plane in panel (a)). Au atoms are shown in yellow, and the reachable positions of the two bottom O_carb_ atoms (see Figure 4) are shown in the color of the respective anchor set. An exemplary molecule in  is drawn in black, with a red sphere marking the corresponding tip position. (c) A tip displacement step (black arrow) causes one anchor atom to change (white arrow in the inset, length exaggerated for better visibility), such that the molecule configuration moves from  to . Note that in panel (c), duplicate anchor sets are displayed, which are rotated and translated with respect to the primary anchor sets shown in panels (a) and (b).

**Figure 7:**
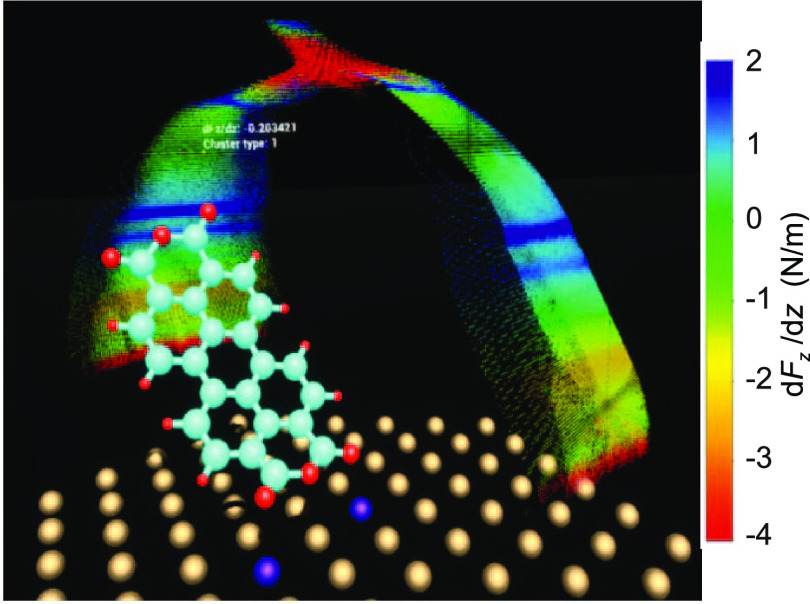
Virtual reality representation of the FSA. Screenshot of the custom-made VR software that allows browsing the FSA and performing simulated manipulations in an interactive manner. Each tip position in the selected anchor set (here ) is represented by a dot, the color of which encodes the force gradient in the respective configuration. Motion capture of the operator hand is used to control the tip position **s** for which the molecular configuration **r** is displayed. Anchor atoms are shown in violet.

The obtained FSA (graph) for PTCDA on Au(111) contains a total of 504.432 states **x** in three anchor sets (branches) and 4.029.635 transitions ***τ***. The tip positions of all states are displayed in Figure 6a for each anchor set separately. The arch-like structure of these positions arises because, in all configurations, the lower oxygen atoms of the molecule bind to their two respective anchor atoms, which thus act like hinges (cf. Figures 2 and 7). As a consequence, the arch of  is rotated by 30° compared to  and  because the respective anchor is rotated likewise (see colored anchor atoms in Figure 6a). The spatial relations between tip positions, anchor atoms, and their reachable areas are depicted in Figure 6b for the tip height *s*_*z*_ = 10 Å. An example of a final state  and the respective transition into the anchor set  is visualized in Figure 6c.

Once the complete FSA has been built, it serves as our observation and state transition model. Providing a state **x** and a tip displacement step *a*_*d*_, we can retrieve the corresponding next state x* = *S*(**x**, *a*_*d*_) and its force gradient F′ = *U*(x*). The latter is obtained by taking the experimental SPM tip oscillation amplitude into account. By discretizing a given experimental tip trajectory into accepted input steps Δ***s***_*j*_ belonging to the input alphabet Σ_1_, we obtain a sequence of molecular configurations **r**_*j*_ and force gradients F_*j*_^′^ for the entire tip trajectory in a stepwise manner. Since this type of query is explicitly supported by the structure of the FSA, it only takes a millisecond to traverse a trajectory consisting of 100,000 states.

We have coupled the FSA to a fully immersive virtual reality display, which allows us to inspect the molecular configuration data interactively in three dimensions (Figure 7). Specifically, we can visualize simulated manipulation processes for which the input, i.e., the desired tip trajectory, is generated by a motion capture device that records the motion of human hands.^23,45^ This interactive exploration of molecular manipulation is crucial since it provides an extremely efficient way to access the large amount of information in the FSA. In this way, an intuition for the manipulation process itself can be developed effortlessly.

#### Information Gathering

In this section, we analyze the FSA regarding the aspect of information gathering. Above, we outlined a concept for calculating the average length *K* (eq 7) that an information-gathering trajectory must have in order to uniquely identify the initial state. Using eqs 6 and 7, we can compute *K* after extracting the required parameters from the FSA.

**Figure 8:**
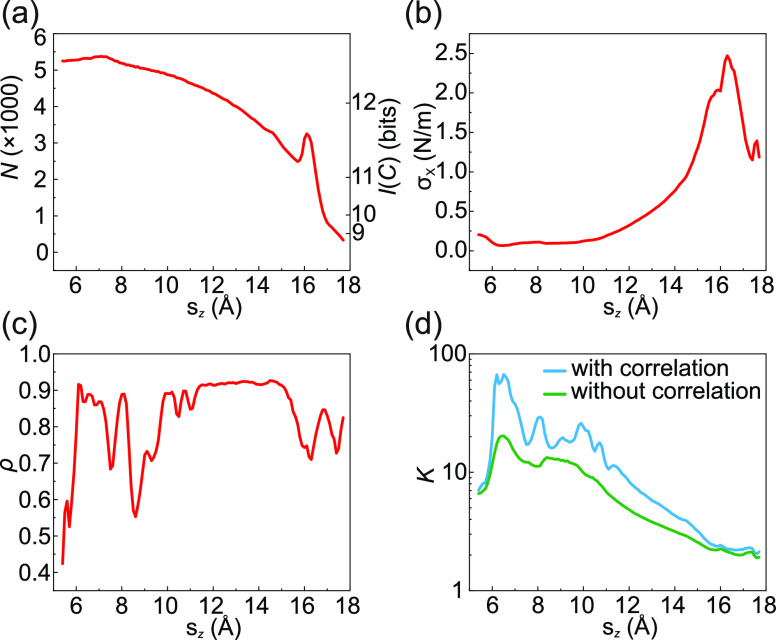
Information gathering. (a) The number of configurations *N* and respective information content *I*(*C*) in Δs_*z*_ = 0. 1 Å wide intervals. (b) Variance σ_*X*_ of the F′ values stored in the FSA. (c) Correlation ρ between the F′ values of all states between which a direct transition exists. (d) The approximate number of steps *K* that would be required to identify a molecular configuration unambiguously with correlation (blue curve) and without correlation (ρ(s_*z*_) = 0, green curve). All quantities are calculated for a series of s_*z*_ intervals of 0.1 Å width.

It is insightful to calculate *K* as a function of tip height instead of giving one number for the entire configuration space . From the FSA, we obtain both the standard deviation σ_*X*_(s_*z*_) of all F′ values and the number of configurations *N*(s_*z*_) in a 0.1 Å wide s_*z*_ interval. Figure 8a reveals that the information required to discern the configurations in a fixed interval around a given value s_*z*_ drops from 12.5 bits to 8.5 bits (8.5 nats to 6 nats) with increasing tip height. In parallel, the distribution of possible F′ values as specified by σ_*X*_ becomes wider as s_*z*_ increases (Figure 8b). For the limiting case of a sequence of uncorrelated F′ values along the information-gathering trajectory, the average number of required steps *K* = *I*(*C*)/*I*(*X*, *Y*) drops from 20 to 2 (green curve in Figure 8d).

While this result describes the tendency correctly, in practice, more steps will be required because the F′ values of neighboring states are typically correlated. As discussed above, we quantify this aspect via the Pearson correlation coefficient ρ, which is obtained from the data in the FSA (Figure 8c). Using eqs 6 and 7, we can now compute *K*(s_*z*_) for the more general case of correlated *X* values. The result is a strong increase in *K* compared to the case of uncorrelated *X* values (Figure 8d). Particularly at smaller s_*z*_ values, up to 60 steps are now required for unambiguous identification of the molecular configuration. For large s_*z*_ values, the effect becomes less dramatic since the variance σ_*X*_ increases and the correlation ρ drops. Having obtained an estimate for the length of a trajectory, the next question to address is that of reversibility.

#### Reversibility

Reversibility is crucial for information gathering since the manipulation process has to return the tip and the molecule to their original state ***x***_0_ once its configuration has been identified. As mentioned above, we expect individual manipulation steps to be reversible if they occur within one and the same anchor set, that is, if the bottom O_carb_ atoms do not change their Au bonding partner. Using the FSA, we are able to investigate reversibility at the fundamental level of FSA transitions ***τ***, which represent individual tip displacements.

**Figure 9:**
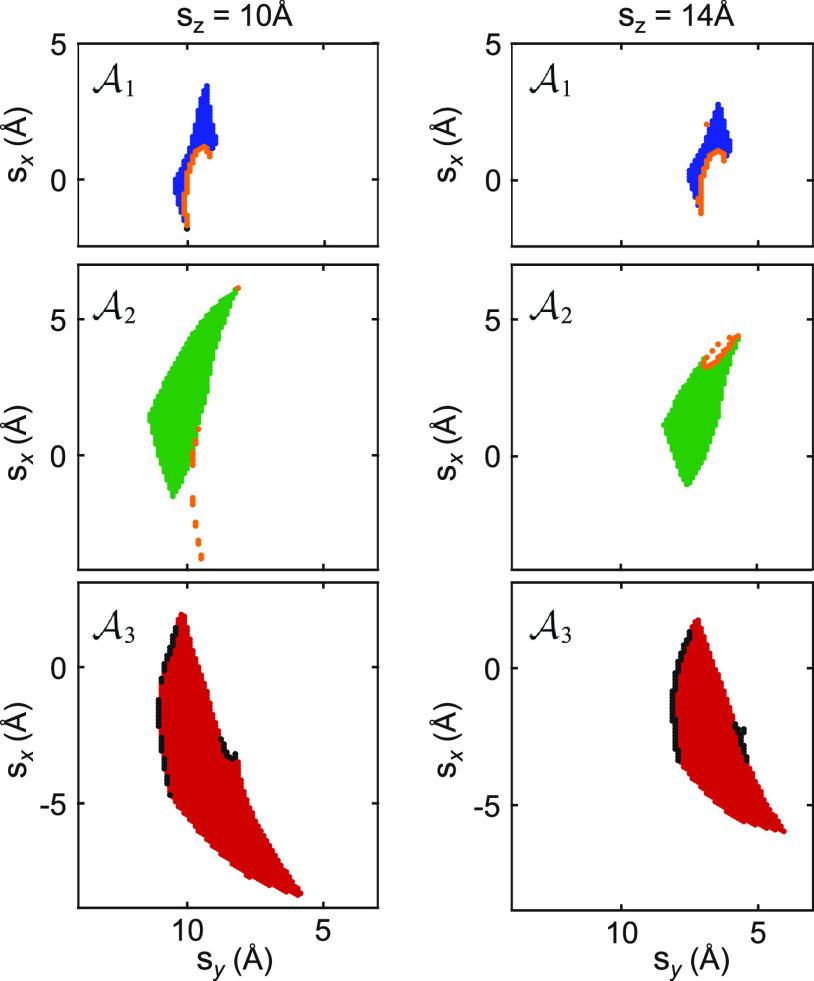
Reversible and irreversible states. Horizontal cuts through the left branch of the **s** clouds of all three anchor sets in Figure 6a at two different heights s_*z*_. The tip positions of states with at least one irreversible transition are colored orange ( and ) or black (). Isolated states (prominent in ) can only be reached from other (duplicate) anchor sets (compare Figure 6c).

We classify each of the approximately 4 × 10^6^ transitions ***τ*** in the FSA as either reversible or irreversible in the following way: a transition τ = (**x**, x*) is reversible if the transition τ′ = (x*, **x**) also exists in the FSA; otherwise, it is irreversible. As a result, we find that only 2.1% of all transitions are irreversible (Tab. 1). In addition, we label each transition τ = (**x**, x*) with respect to its connectivity as either an external transition, if it connects states **x** and x* in two different anchor sets (including symmetry equivalents), or as an internal transition, if it connects states within the same anchor set. Combining the two properties of connectivity and reversibility, as done in Table 1, we find that 99.8% of the internal transitions, but only approximately 75% of the external transitions, are reversible. Thus, we can conclude that a reconfiguration of the Au–O_carb_ bonds at the lower end of the molecule indeed has a 25% chance of introducing irreversibility into the molecular manipulation process. In Figure 9, this has been visualized: the lateral tip positions (s_*x*_, s_*y*_) of states, which have at least one irreversible (outgoing) transition, are practically always located on the outer hull of their anchor set. Irreversibility within an anchor set (found for 0.2% of all internal transitions) can, for example, result from an abrupt inversion of the bending direction of PTCDA: while being concave when lifted (Figure 1a), it might bend away from the surface in a convex metastable configuration when lowered under tension from the vertical. Finally, we note that a different atomistic simulation approach may influence the precise percentages in Table 1 (see Methods), but the general conclusion regarding reversibility is expected to be robust.

**Table 1:** Percentage of FSA Transitions Which Belong to the Reversible/Irreversible and Internal/External (Connectivity) Categories

	external	internal
reversible	5.6%	92.3%
irreversible	1.9%	0.2%

With the results of Table 1 and Figure 9, we have obtained the simple rule of thumb that all states within a single anchor set can safely be visited for information gathering in order to identify a given initial state ***x***_0_. Note, however, that as long as the boundaries of individual anchor sets are not known in the experiment, this rule is only of limited value. In the Supporting Information, we analyze the reversibility of full manipulation trajectories in a simulated experiment. We find that for the given molecule–surface system, the chances of returning to a state other than ***x***_0_ increase at a rate of approximately 2.5% per Angstrom trajectory length. Information gathering must therefore be balanced between the amount of obtainable information and the risk of irreversibility, both of which increase with trajectory length.

### Particle Filter

#### Objectives

With the basic functionality of the PF outlined above, we formulate the following two objectives for its practical application in configuration monitoring:

1. In a manipulation process in which, starting from the initial configuration ***x***_0_ with observation *F*_0_^′^, *M* action-observation pairs (Δ***s***_*j*–1_, *F*_*j*_^′^) are recorded for *j* = 1, ···, *M* sequentially, we want to determine the best *ad hoc* estimate **x̃**_*j*_ for the configuration ***x***_*j*_ (which yields the observation *F*_*j*_^′^) at each step *j* of the trajectory.
2. Further, we want to determine the consistent manipulation trajectory **x̌**_*j*–1_ with *j* = 1, ···, *M* + 1 that agrees best with *F*_0_^′^ and the entire sequence *j* = 1, ··· *M* of action-observation pairs (Δ***s***_*j*–1_, *F*_*j*_^′^).

In the second objective, the term consistent means that the sequence of configurations **x̌**_0_ ··· **x̌**_*M*_ should evolve from the initial configuration **x̌**_0_ as dictated by the individual tip displacement steps Δ***s***_*j*–1_, *j* = 1, ···, *M*, and the state transition model *S* as **x̌**_*j*_ = *S*(**x̌**_*j*–1_, Δ***s***_*j*–1_). In contrast, the sequence **x̃**_0_ ··· **x̃**_*M*_ of the best ad hoc configurations from objective (1) does not have to fulfill this consistency requirement, such that subsequent configurations **x̃**_*j*–1_ and **x̃**_*j*_ do not necessarily have to be related at all. This distinction between **x̃** and **x̌** is necessitated by the probabilistic nature of the PF (see below) and the fact that, given the limited available information that is particularly sparse at the beginning of the manipulation trajectory, several entirely different regions in the configuration space  can provide good estimates for ***x***_*j*_. Consequently, the PF may locate **x̃**_*j*–1_ and **x̃**_*j*_ in two different regions of , between which Δ***s***_*j*–1_ does not provide a transition, i.e., **x̃***_j_* ≠ *S*(**x̃**_*j*–1_, Δ***s***_*j*–1_), while for the consistent trajectory, we enforce a sequence of configurations **x̌**_*j*_ which (in the spirit of eq 5) follows deterministically from **x̌**_0_ and the experimental tip translation steps Δ***s***_*j*–1_. Specifically, when determining the consistent trajectory, all measured data points *F*_0_^′^, ··· *F*_*M*_^′^ are taken into account on an equal footing, such that even the value *F*_*M*_^′^ obtained at the very end of the manipulation process contributes to the determination of the configuration **x̌**_0_ at its very beginning.

Since the above objectives exceed the typical functionality of a PF, we will first describe the basic operation principle and algorithm of a PF and subsequently discuss the additionally required modifications to achieve configuration monitoring.

#### Operation Principle

**Figure 10:**
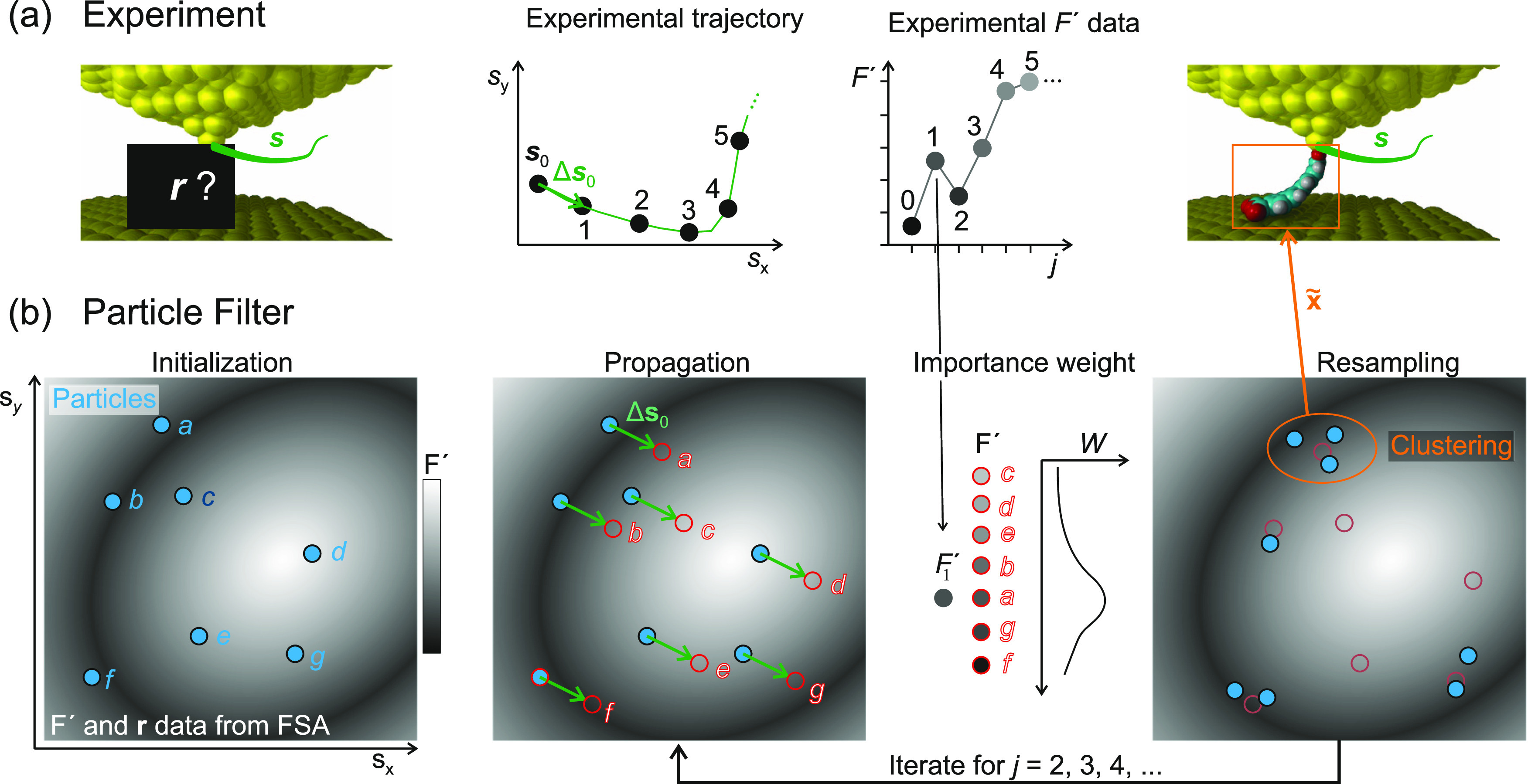
Operation principle of the particle filter. (a) Experiment. Starting from an unknown molecule configuration ***r***_0_, the tip is moved along a trajectory ***s***_*j*–1_, *j* = 1, ···, 5 in the *x*,*y* plane (green), and force gradients *F*′ (***s***_*j*–1_) are recorded. (b) Particle filter. Initialization. Particles (*G* = 7) are dispersed in  at random tip–molecule configurations **x**_*l*_, *l* = 1, ···, 7 (blue). The gray background symbolizes the observation model F′ = *U*(*x*) stored in the FSA. (1) Propagation. All particles are displaced according to the experimental tip displacement step Δ***s***_0_ and the state transition model as **x**_*l*,1_ = *S*(**x**_*l*,0_, Δ***s***_0_). Synthetic noise in the displacement is omitted here. Each particle *l* has a distinct F_*l*_^′^ (background greyscale). (2) Importance weight. According to the agreement between their F_*l*_^′^ value and the experimental *F*′, the particles receive individual importance weights *W*_*l*_ (eq 9). (3) Resampling. All particles are randomly relocated to the proximity of previous particle locations (faint red), favoring the original locations of particles with high *W*_*l*_ (here: particles *a*, *b*, *g*, and *f*). Exploration places a fraction ϵ of the particles in completely random locations (not shown). (4) Clustering. Regions with high particle density are identified because they represent the PF’s best estimates of the actual molecular configuration ***r***_1_, which is the property of interest. The PF will iterate through steps (1)–(3) for *j* = 2, 3, ···, converging the particle locations further onto good configuration estimates for ***x***_*j*_. Step (4) is only required when an ad hoc conformation estimate **x̃** is requested.

The PF neither provides a probability distribution across all states in  nor a single most promising state estimate **x̃**_*j*_, but a set of *G* more or less likely estimates of the current experimental configuration ***x***_*j*_. These estimates are the set of particles . At each step *j*–1 of the PF, the prior information gained from *F*_0_^′^ and all action-observation pairs (Δ***s***_0_, *F*_1_^′^), ···, (Δ***s***_*j*–2_, *F*_*j*–1_^′^) is contained exclusively in the distribution of these particles in . To update the prior according to the action Δ***s***_*j*–1_ in step *j*, the entire particle cloud **x**_*l*,*j*–1_ is relocated according to the state transition model to **x**_*l*,*j*_ = *S*(**x**_*l*,*j*–1_, Δ***s***_*j*–1_), ∀*l*. Then, the particles are redistributed such that a higher particle density is created in regions of state space where particles have just been evaluated comparatively positively (Figure 10). In our case, this means a good correspondence between simulated and measured force gradient values F_*l*,*j*_^′^ and *F*_*j*_^′^ at this step. A high local density of the particle cloud thus indicates a cluster of estimates which have performed particularly well over the last couple of iterations. The rate at which particles agglomerate, as well as other hyperparameters of the PF, have to be adjusted to the problem at hand. The stepwise operation of the PF is preceded by an initialization in which the *G* particles are randomly placed in . If available, prior knowledge, such as an approximate height of the tip or an approximate azimuthal angle of the molecule in the experiment, can be included by biasing the initial distribution of sampling points (particles) accordingly.

#### Algorithm

In detail, at each step *j*, the PF performs the following three actions to update its estimate of the molecule configuration ***x***_*j*_ in the experiment (Figure 10):

(1) Propagation. The particles are propagated using the tip translation step Δ***s***_*j*–1_ and the state transition model stored in the FSA. This propagation forces each particle to replicate the movement of the tip and to transform its configuration from **x**_*l*,*j*–1_ to **x**_*l*,*j*_ accordingly (see above). If one of the particles **x**_*l*,*j*–1_ indeed matched the experimental molecular configuration ***x***_*j*–1_, the propagation step ensures that also **x**_*l*,*j*_ and ***x***_*j*_ match. If the length of the tip translation step Δ***s***_*j*–1_ exceeds the nearest-neighbor distance in the dense discretization grid of the FSA, **x**_*l*,*j*–1_ and **x**_*l*,*j*_ will be FSA states between which no direct (one-step) transition exists. In this case, a path-finding algorithm^46^ is used to calculate the required intermediate transitions. Subsequently, each particle receives an additional random displacement which scales with the length of Δ***s***_*j*–1_ but never exceeds three FSA transitions. This randomization accounts for the fact that a certain degree of stochasticity is present in the experiment, even when performed at cryogenic temperatures.

(2) Importance weight. The second step is the calculation of an importance weight *W*_*l*,*j*_ for each particle *l*. It quantifies how well the simulated F_*l*,*j*_^′^ value of the particle matches the measured value *F*_*j*_^′^ at this step. As discussed above, the importance weight approximates *P*(**x**_*l*,*j*_|*F*_*j*_^′^) in eq 5, defined as the conditional probability that the configuration is **x**_*l*,*j*_ if *F*_*j*_^′^ is measured. Here, it is assumed that the probability that the specific configuration **x**_*l*,*j*_ of particle *l* matches the actual configuration ***x***_*j*_ in the experiment increases with the importance weight *W*_*l*,*j*_ of **x**_*l*,*j*_. We calculate the importance weight as
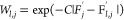
9where *C* is the constant importance weight factor, one of the hyperparameters of the PF.

(3) Resampling. Finally, the particles are redistributed. A small fraction ϵ ≪ 1 of all particles is randomly placed in , with the exploration factor ϵ being a hyperparameter of the PF. All other (1 – ϵ) *G* particles are relocated into the proximity of states for which large importance weights have been computed in (2). Since placement probabilities scale with the respective importance weights *W*_*l*,*j*_ of each location (see Methods), configurations with high *W*_*l*,*j*_ will accumulate particles in their vicinity, that is, within a (randomly chosen) distance of up to three neighboring FSA states for the purpose of exploration. This updates the points at which the configuration space  is sampled in the following trajectory step *j* + 1. The agglomeration of particles in promising regions of the configuration space updates the prior information for this following step, similar in spirit to the repeated use of eq 4 (or a single evaluation of eq 5).

Three hyperparameters determine the behavior of the PF:

- The number of particles *G* used for sampling the configuration space . More particles result in a higher chance of finding a configuration that matches the unknown configuration in the experiment.
- The exploration factor ϵ ≪ 1 defines the fraction of particles that are randomly initialized during each step instead of being relocated to the vicinity of a particle position from the previous generation of particles.
- The importance weight factor *C* is a multiplier in the importance weight function (eq 9), which is used to control the convergence of the PF. Large *C* values mean fast convergence onto the most promising particle positions.

#### Configuration Estimation with the PF

The iterative procedure described above covers the basic functionality of the PF. However, to obtain an estimate of the current most likely configuration **x̃**_*j*_, as desired for objective (1) above, further steps are required. To obtain **x̃**_*j*_, we have to identify regions with a particularly high particle density. Since particles with large (relative) importance weight *W*_*l*_ attract more particles to their vicinity, a considerable number of particles will accumulate in regions of  where they maintain a large *W*_*l*_ over several PF iterations. Unlike any single particle which happens to have the largest *W*_*l*,*j*_ in a particular step *j*, such clusters indicate groups of particles with a high prior probability, that is, particles that have performed particularly well in the past and that will typically be very similar in terms of their tip–molecule configurations **x** and force gradients F′. In this case, the entire cluster represents a single good estimate of the experimental molecular configuration ***x***_*j*_.

To identify **x̃**_*j*_, we search, at the respective step *j*, for the largest cluster of particles in the entire particle distribution. To this end, we use the K-medoid clustering technique^28^ (see Methods), which splits the complete set of particles  into *K* subsets . Following the arguments above, we assume the largest cluster, as a whole, to represent the best estimate for ***x***_*j*_. Since a specific configuration **x̃**_*j*_ has to be determined, we retrieve the medoid (center) particle as a representative member of that cluster. The computational effort for the clustering is quadratic in the number of particles *G* and, therefore, typically more demanding than the plain PF, which scales linearly with *G*. Hence, one can seek a trade-off between accuracy (more particles) and frequent updates of the configuration estimate **x̃** by clustering.

As pointed out above, configuration estimates **x̃**_*j*–1_ and **x̃**_*j*_ of consecutive steps may turn out to be completely unrelated to each other, such that the sequence of all **x̃**_*j*_, with *j* = 0, ···*, M* will typically not represent a valid manipulation trajectory: the tip–molecule configuration **x̃**_*j*_ instead varies discontinuously with *j*, as exemplified in Figure 12 and in two supplementary animations. As mentioned in objective (2) above, a solution to this problem is the computation of a consistent manipulation trajectory **x̌**_*j*_, *j* = 0, ··· *M*. We select promising consistent trajectories using a greedy algorithm inspired by beam search.^47^ Specifically, we calculate *M* consistent trajectories **x̌**_*j*,γ_, with γ = 1, ···, *M*, based on the previously found *M* ad hoc configurations **x̃**_*j*_, *j* = 1, ···, *M*, which are considered to be good configuration estimates. To this end, we propagate each **x̃**_*j*_ forward and backward in time, using the state transition model and the sequence of tip steps Δ***s***_0_, ···, Δ***s***_*M*–1_, such that each consistent manipulation trajectory by construction passes through one of the configurations **x̃**_*j*_; for the consistent trajectory γ, this crossing occurs at **x̌**_γ__,γ_ = **x̃**_γ_. Subsequently, we select the consistent trajectory that best matches the experimental *F*′ values. The respective deviation is computed over the entire length of each trajectory as
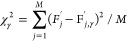
10Based on their χ^2^ values, we can judge which of the underlying configurations **x̃**_*j*_ was indeed a good estimate and which only performed well on a small segment of the entire set of action-observation pairs (Δ***s***_*j*–1_, *F*_*j*_^′^). Note that the best estimate for the consistent trajectory is not necessarily found by the PF close to γ = *M* because, due to simulation uncertainties, noise, and the stochasticity of its operation principle, the PF can, in spite of the correct propagation (1), loose a once found good estimate as the manipulation progresses. In the following, we will, for simplicity, denote the best consistent trajectory **x̌**_*j*,γ_ with the lowest χ_γ_^2^ as **x̌**_*j*_.

### Results of PF Application

We now continue with benchmarking the PF by applying it to synthetic as well as experimental data sets from refs (8, 17).

#### PF Performance on Synthetic Data

**Figure 11:**
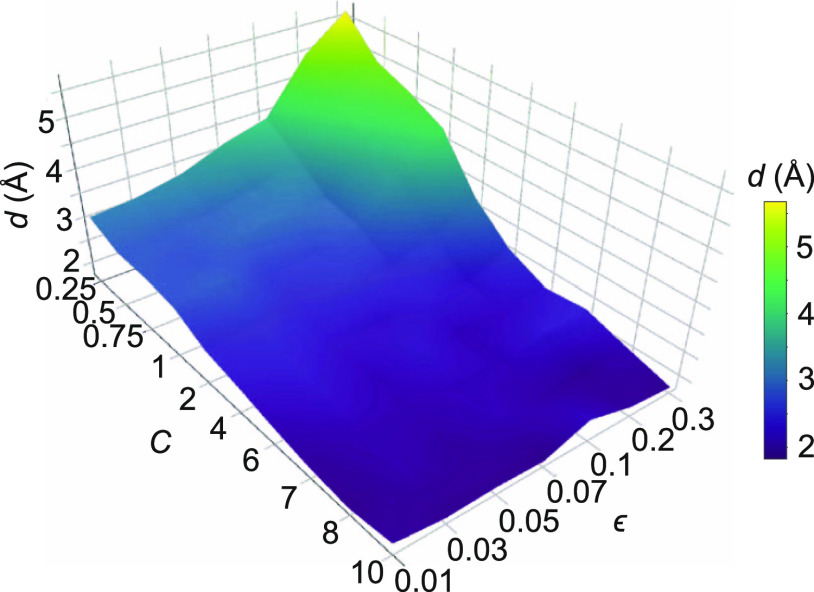
Grid search over PF parameters. The plot shows the average distance *d* (eq 11) between the predicted and the actual (ground truth) tip positions s̃_*j*_ and *s* for PF runs with various combinations of *C* and ϵ.

We first use synthetic (***x***_*j*_, *F*_*j*_^′^) trajectories sampled from the FSA to assess the performance of the PF for various combinations of hyperparameters. Note that in this case, ground-truth molecular configurations ***r***_*j*_ are available. Since vertical tip trajectories with fixed (*s*_*x*_, *s*_*y*_) were used in the experiments that will be analyzed in the following section, we also limit the synthetic data to vertical tip trajectories. We assess the quality of each configuration estimate **x̃**_*j*_ by comparing the actual tip positions, ***s***_*j*_, with the predicted ones, **s̃**_*j*_, using the distance
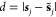
11as a measure of the PF performance (Figure 11). We note that the azimuthal angles of the molecule are implicitly also compared to the ground-truth data because ***s***_*j*_ and **s̃**_*j*_ are defined relative to the mid-point between the two anchor atoms of the corresponding anchor, thus effectively including information on the azimuthal angle in the tip position.

To optimize the hyperparameters of the PF, we create a data set of 10 vertical tip trajectories sampled from the FSA at random lateral tip positions (*s*_*x*_, *s*_*y*_) and random azimuthal orientations of the molecule. We introduce noise to the synthetic *F*′ data by randomly offsetting the tip at each point of the trajectory by up to three steps in every direction of the FSA (that is, by about 0.3 Å) and choosing the *F*′ value at this location. While the number of particles (*G* = 1500) that we employ is dictated by the required speed of the PF, the exploration rate ϵ and the importance weight factor *C* can be chosen freely. We explore these parameters in a grid search with the values ϵ ∈ {0.01, 0.02, 0.05, 0.07, 0.1, 0.2, 0.3} and *C* ∈ {0.25, 0.5, 0.75, 1, 2, 4, 6, 7, 8, 10}. Since the PF is stochastic in nature, we have performed 10 PF runs with different random initializations on each vertical trajectory, resulting in a total of 100 different tests for each of the 70 different parameter combinations.

The result of the performance evaluation is displayed in Figure 11, where we plotted the distance *d* (eq 11) averaged over all *M* points of each trajectory and over the 100 PF runs at each (ϵ, *C*) combination. It shows that the PF performs poorly if set to slow convergence and high exploration. In this regime, the particles remain almost entirely randomly distributed throughout all steps of the PF. This renders the outcome of the K-medoid clustering and thereby **x̃** completely arbitrary, which explains the poor PF performance. In contrast, for *C* ≥ 5 and ϵ ≤ 0.1, the PF performs well. For the application of the PF to experimental data, we, therefore, select *C* = 5 and ϵ = 0.05, which are values well inside the range of our grid search (Figure 11).

**Figure 12:**
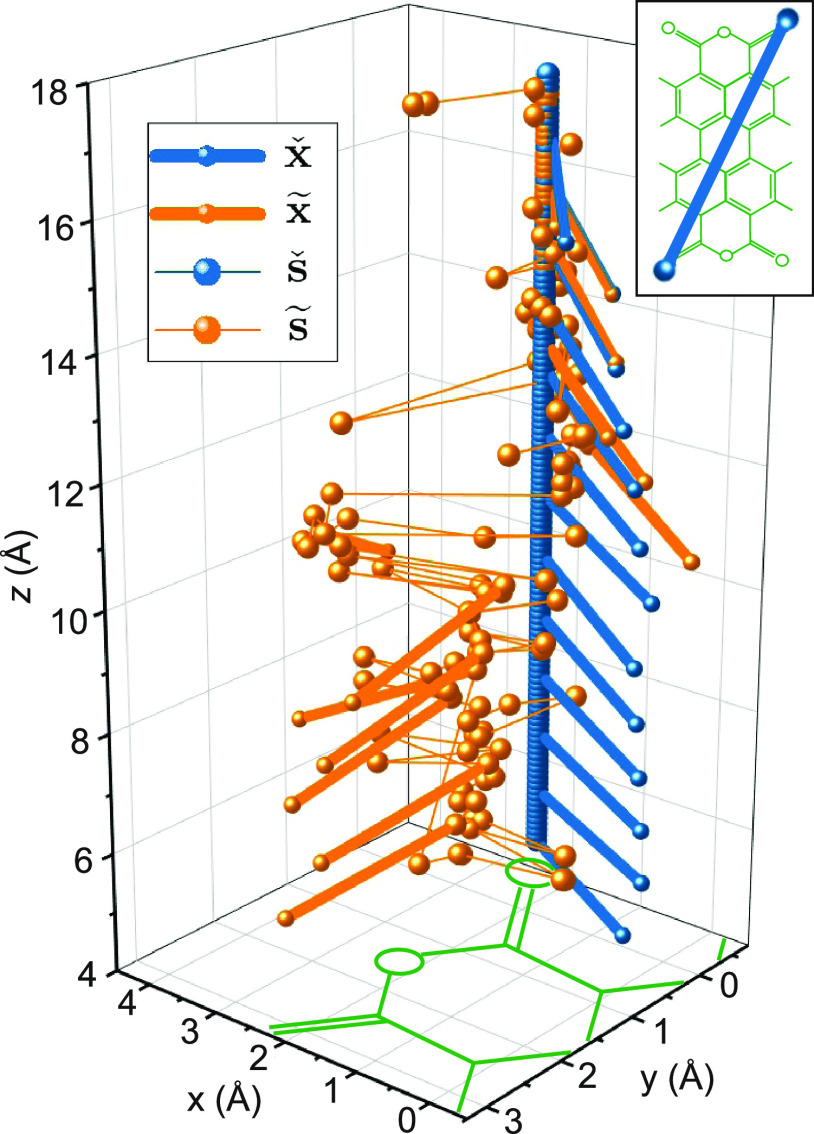
Ad hoc and consistent trajectories. Example of the **x̃**_*j*_ and **x̌**_*j*_ configuration estimates in a PF run on a synthetic data set that starts at low *s*_*z*_ values. Large spheres mark tip positions, thin lines connect subsequent **s̃**_*j*_ points, and thick lines and small spheres visualize the azimuthal orientation of the molecule (see inset) for selected *j* values. The molecule (green) at the bottom of the main panel is drawn to scale.

In Figure 12, we visualize the qualitative difference between the two configuration estimates **x̃**_*j*_ and **x̌**_*j*_, using one of the synthetic data sets as an example. As expected, the tip position **s̃** and, with it, the azimuthal molecular orientation often jump erratically between subsequent configurations **x̃**_*j*_ and **x̃**_*j*+1_, particularly in the first half of the trajectory (*s*_*z*_ ≲ 11 Å). In contrast, the consistent trajectory exhibits a constant lateral tip position and a molecular orientation that evolves smoothly. In fact, the consistent trajectory **x̌** matches the ground-truth configuration ***x*** perfectly, which was therefore omitted from the plot. Finally, the plot shows that the ad hoc estimates **x̃** improve substantially and become more consistent during the manipulation process; this is to be expected as more *F*′ data becomes available and the particles have the chance to cluster in configuration space . The analysis of information gathering above revealed that 20–60 steps are required to identify a configuration unambiguously at *s*_*z*_ < 10 Å, while (on average) only 2 steps are required at large tip height. This is consistent with the better performance of the FSA at larger *s*_*z*_ in Figure 12.

**Figure 13:**
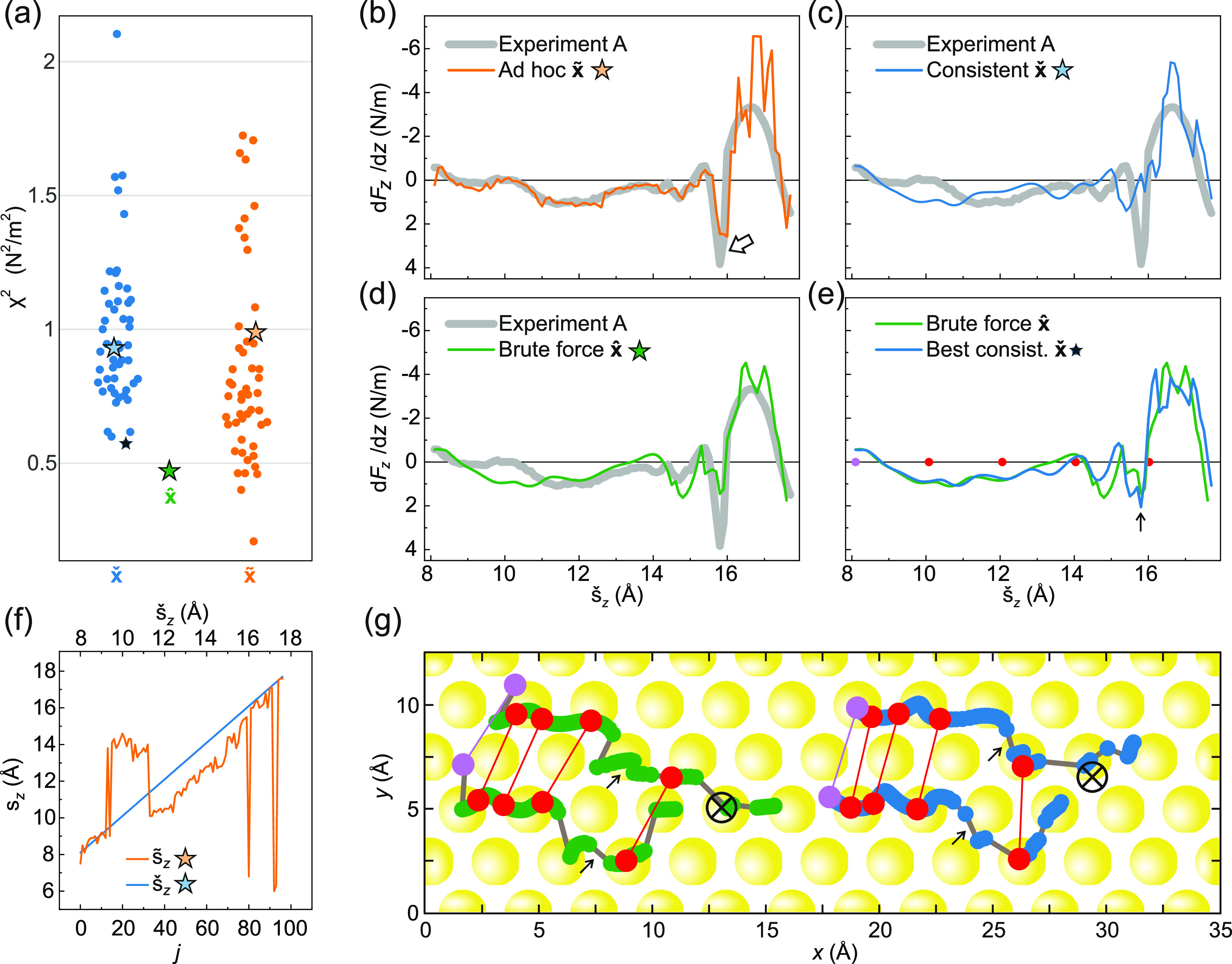
Ex-post configuration monitoring of experiment A. (a) Deviation χ^2^ (eq 10) between *F*′ (*s*_*z*_) on the one hand and F̌_*j*_^′^ (blue) or F̃_*j*_^′^ (orange) on the other, for 50 PF runs. The χ^2^ value for the best possible consistent trajectory (brute-force search) is shown in green. Blue and orange stars mark χ^2^ values for the PF run with the median χ^2^ value for F̌′(s_*z*_). The black star marks the run with the lowest value for F̌′(s_*z*_). (b)–(e) Comparison of the experimental *F*′(*s*_*z*_) curve (gray) and exemplary PF results. Panels (b) and (c) show F̃_*j*_^′^ and F̌_*j*_^′^ curves with the median χ^2^ value from panel (a). Panels (d) and (e) show F̂_*j*_^′^ compared to the experiment and to the best F̌_*j*_^′^ curve found by the PF [black star in panel (a)]. (f) The plot of predicted tip height for the ad hoc and the consistent PF results, s̃_*z*,*j*_ and š_*z*,*j*_. (g) Traces of the two bottom O_carb_ atoms of the brute-force trajectory **x̂**_*j*_ (green) and the best consistent trajectory **x̌**_*j*_ (blue) found by the PF. Initial positions are color-coded in pink, and subsequent positions at specific s_*z*_ heights are in red. The lateral tip position is marked by an encircled cross (black), and O_carb_ atom jumps related to specific features in the F′(s_*z*_) curves (see the text) are marked by black arrows.

**Figure 14:**
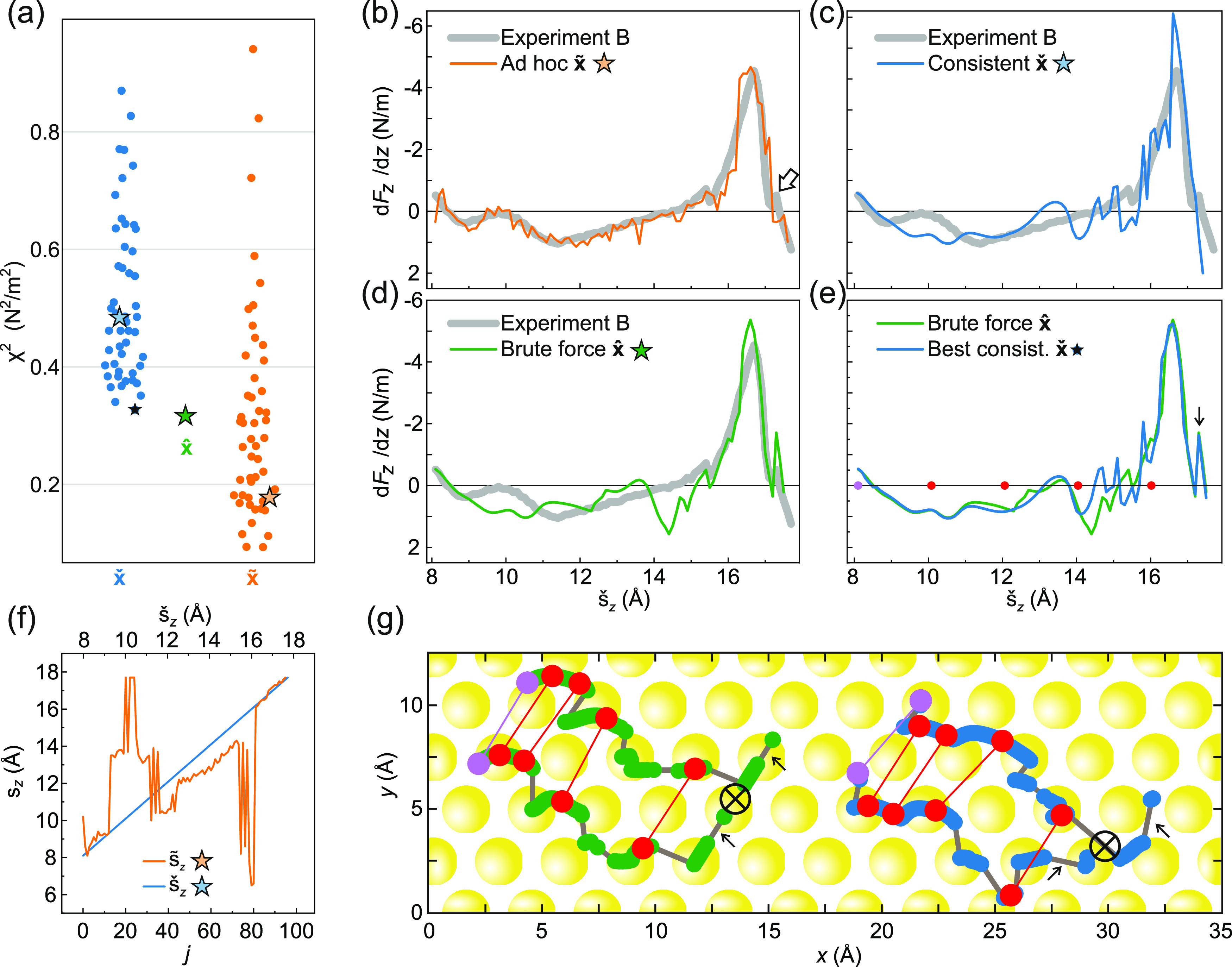
Ex-post configuration monitoring of experiment B. For a detailed description of the panels, see Figure 13. (a) The χ^2^ plot for F̌_*j*_^′^ (blue) and F̃_*j*_^′^ (orange). (b)–(e) Comparison of *F*′(*s*_*z*_) from experiment B to F̃_*j*_^′^, F̂_*j*_^′^ and F̌_*j*_^′^. (f) Predicted tip heights s̃_*z*_, and š_*z*_. (g) Traces of both bottom O_carb_ atoms of the brute-force trajectory **x̂**_*j*_ and the best consistent trajectory **x̌**_*j*_.

The speed of the PF can be tuned in a wide range by adjusting the number of particles *G* and the frequency of K-medoid clustering, which requires 90% of the computational effort at *G* = 1500. In the present example, we clustered after each PF step. This, however, would usually not be necessary. In a plausible scenario, one could perform clustering steps less frequently and instead increase the number of particles, thus keeping the PF speed constant. More particles allow a denser sampling of , which would, on average, improve the quality of the obtained configuration estimates and reduce the random scattering of the quality between individual PF runs (see also Figures 13a and 14a). On the downside, however, this would also reduce the frequency of explicit ad hoc configuration estimates **x̃** (by clustering) and thus also the number of configurations from which a consistent trajectory **x̌** can be constructed.

#### Retrieving Molecule Configurations from Experimental Data

Having optimized its hyperparameters on synthetic data, which provide the ground truth as a benchmark, we now apply the PF to actual experiments. Specifically, we selected two vertical lifting experiments from ref (8). As Figure 1b reveals, these two experiments exhibit distinctly different *F*′(*s*_*z*_) curves. For each of the two, we perform and analyze 50 PF runs. Here, each run represents a possible outcome of a hypothetical scenario in which the PF is used in real time alongside the experiment. With *G* = 1500 particles, the speed requirements for such an application can indeed be met since the execution of a single PF step takes approximately 1.8 s. To offer an unbiased impression of the PF performance, we have not considered any prior information in our examples.

Testing the PF on experimental data is conceptually different from tests on synthetic trajectories because for experimental data, i.e., action-observation pairs (Δ***s***_*j*–1_, *F*_*j*_^′^), the ground-truth configurations ***x***_*j*_ are not known. We can, therefore, only assess the capability of the PF to reproduce the individual experimental *F*_*j*_^′^ → *F*′(*s*_*z*_) curves as well as possible. Importantly, the capability of any search method aimed at finding the correct experimental trajectory is intrinsically limited by the observation and the state transition models. If the models cannot reproduce parts of the measured *F*′(*s*_*z*_) data for any configuration , no search algorithm will be able to match the *F*′(*s*_*z*_) curve in its entirety. This turns out to be the case in the examples discussed below.

We determine the discrepancy between the experimental and the simulated force-gradient curves with the help of eq 10 and *M* = 96 individual tip translation steps per curve. To obtain a reference against which the PF can be benchmarked, we additionally perform an FSA-based brute-force search across all *N* conceivable manipulation trajectories **x**_*i*,0_··· **x**_*i*,*M*_, *i* = 1 ··· *N*, each of which starts at a different configuration  and follows the experimental tip displacement steps Δ***s***_0_··· Δ***s***_*M*–1_. Since the tip is retracted vertically in the experiments analyzed here, those steps are simply Δ*s*_*z*_ translations of constant length. The brute-force search requires 3.5 h, while a PF run takes about 3 min.

The results of the 50 PF runs for experiments A and B are summarized in Figures 13 and 14, respectively. Each PF run yields two sequences of configurations **x̃**_*j*_ and **x̌**_*j*_, with *j* = 0, ··· 96, both of which have their associated F̃_*j*_^′^ and F̌_*j*_^′^ sequences. Examples of the latter are compared to the experimental *F*′ curve in panels (b) and(c), while some properties of the configurations **x̃**_*j*_ and **x̌**_*j*_ are presented in panels (f) and (g). Finally, the brute-force search yields a sequence **x̂**_*j*_ with its associated F̂_*j*_^′^ (panels (d) and (e)). Figures 13a and 14a show that the χ^2^ values (eq 10) of both the F̃_*j*_^′^ and F̌_*j*_^′^ sequences scatter substantially, varying by almost a factor of four.

While only the best consistent trajectories **x̌**_*j*_ (blue) come close to the χ^2^ values of the brute-force trajectories **x̂**_*j*_ (green), it is remarkable that many of the χ^2^ values for the ad hoc configurations **x̃**_*j*_ (orange) are smaller than the brute-force value, in particular for experiment B. This is a consequence of our specific molecular mechanics observation model, which cannot fully reproduce the measured *F*′(*s*_*z*_) curves. Hence, even the best possible consistent simulation (brute force) must have a χ^2^ well above zero. The sequence of ad hoc configurations **x̃**_*j*_, on the other hand, may also yield impossible manipulation trajectories in which both the tip height and the azimuthal molecular orientation jump discontinuously. This becomes clear when the tip heights of one selected ad hoc and one selected consistent trajectory are compared in Figures 13f and 14f. By construction, š_*z*,*j*_ increases by Δ*s*_*z*_ for each step *j* → *j* + 1, but s̃_*z*,*j*_ exhibits several large jumps. Particularly for the (consistent) tip heights in the range 9.5 < *s*_*z*_ < 11.5 Å (15 < *j* < 35), the simulation is apparently not capable of reproducing the measured *F*′(*s*_*z*_), which are in fact close to zero in this region. The PF, therefore, returns configurations **x̃**_*j*_ with tip heights s̃_*z*,*j*_ ≥ 13 Å, for which F′ values close to zero are indeed possible in the simulation, but disregarding the fact that these values of s_*z*,*j*_ are incompatible with the overall manipulation process. It is likely that this effect contributes to the substantial performance variations between different PF runs that we observe in Figures 13a and 14a because, tracing the entire *F*′(*s*_*z*_) curve, the best solutions were found in disparate particle clusters between which the PF had to jump. Importantly, this is no deficiency of the PF but expresses its expected (and desired) flexibility. This example, in retrospect, also confirms the necessity to extend the PF to the computation of the best consistent trajectory **x̌**_*j*_.

To offer a realistic impression of the average performance of the PF, we choose the PF run with the median χ^2^ value for F̌′(s_*z*_) and plot the obtained F̌_*j*_^′^ and F̃_*j*_^′^ sequence in panels (b) and (c) of Figures 13 and 14 (blue and orange stars). To illustrate also the best PF performance, the F̌_*j*_^′^ sequence with the lowest χ^2^ value (black star) is compared to the brute-force sequence F̂_*j*_^′^ in panel (e). For both experiments A and B, the comparisons show that F̌_*j*_^′^ sequences with low χ^2^ values capture all the characteristic features of the experimental *F*′(*s*_*z*_) curves to the same degree as they are reproduced in the brute-force solution. This is reassuring with regard to the real-time application of the PF for the control of a live experiment. In such a scenario, a brute-force search would not be possible.

In the final step of our analysis, we turn to salient features of the predicted tip–molecule configurations **x̌**_*j*_ and **x̂**_*j*_ themselves. As mentioned before, the positions of both the SPM tip and the lower O_carb_ atoms relative to the surface lattice largely define the manipulation process. Therefore, we plotted the lateral paths of the two lower O_carb_ atoms across the Au(111) surface in Figures 13g and 14g for **x̂**_*j*_ (green) and **x̌**_*j*_ (blue). For the latter, the trajectory with the lowest χ^2^ (black star) was chosen. In these plots, the O_carb_ positions are shown as filled circles for each of the points *j* = 0, ···, *M*, with pink and red symbols representing reference positions at *j* = 0, 20, 40, 60, 80 that are also highlighted by markers in panel (e). The tip position is indicated by an encircled cross. As expected from the model of a nearly stiff molecule,^10,18^ the bottom oxygen atoms initially move slowly on the surface until the molecule is oriented almost vertically, whence a small change in the tip height leads to a strong lateral shift of the O_carb_ positions. Our results also show that the O_carb_ atoms prefer sites close to on-top positions, between which they tend to jump abruptly, particularly in the second half of the lifting process when the molecule is inclined substantially. These jumps are both a manifestation and a confirmation of the anchor concept that we employed in our FSA.

A careful analysis of the O_carb_ trails on the surface proves the eminent role of local Au–O_carb_ bonds. We exemplify this with the help of three prominent features in the experimental *F*′(*s*_*z*_) curves, namely, the sharp dip found in experiment A at *s*_*z*_ = 15.8 Å, the small spike in experiment B at *s*_*z*_ = 17.3 Å (marked by open arrows in Figures 13b and 14b, respectively), and the differences in height and shape of both main *F*′ peaks around *s*_*z*_ = 16.7 Å. Since these features are also found in the respective F̂_*j*_^′^ (green) and F̌_*j*_^′^ (blue) sequences in panel (e), their origin can be analyzed on the basis of **x̂**_*j*_ and **x̌**_*j*_—a first showcase of configuration monitoring as proposed in this work (albeit still ex-post). Configuration monitoring indeed reveals that both sharp dip and sharp spike are directly preceded by an abrupt relaxation of both O_carb_ atoms together. The respective jumps are marked by black arrows in Figures 13g and 14g and in the F̌_*j*_^′^ and F̂_*j*_^′^ plots in Figures 13e and 14e; they are also visible in the visualizations of the lifting process (Supporting Information).

Unlike the spike and dip, the main peak at *s*_*z*_ = 16.7 Å is a generic feature of the force gradient curves *F*′(*s*_*z*_) in all experiments in which the molecule is removed from the surface. Overall, the potential energy curve of the molecule on the surface exhibits two rounded steps to lower binding energies,^10^ one connected with overcoming the extended bond of the flat-lying molecule to the surface (i.e., peeling the molecule off the surface), the other associated with overcoming the local O_carb_–Au bonds at the bottom of the molecule (i.e., finally detaching the molecule from the surface). The main peak in *F*′(*s*_*z*_) occurs at the point of maximum potential curvature after the first plateau when, in the wake of breaking the extended bond, the attack on the local bonds begins. At the zero crossing of the *F*′(*s*_*z*_) curve to the right of the force gradient peak (around *s*_*z*_ = 17.7 Å), the slope of the potential reaches its maximum, indicating that the O_carb_–Au bonds are essentially broken. Thus, the main peak in the force gradient curve at around *s*_*z*_ = 16.7 Å is the signature of finally detaching the molecule from the surface. Its specific shape encodes how precisely this process proceeds down to the atomic level. We have in the past observed a great variability of its peak shape,^16^ and also, in the present experiments A and B, they look markedly different. In experiment B, the peak appears sharp, while in experiment A, it is broader, less intense, and more flat-topped (cf. also Figure 1b).

**Figure 15:**
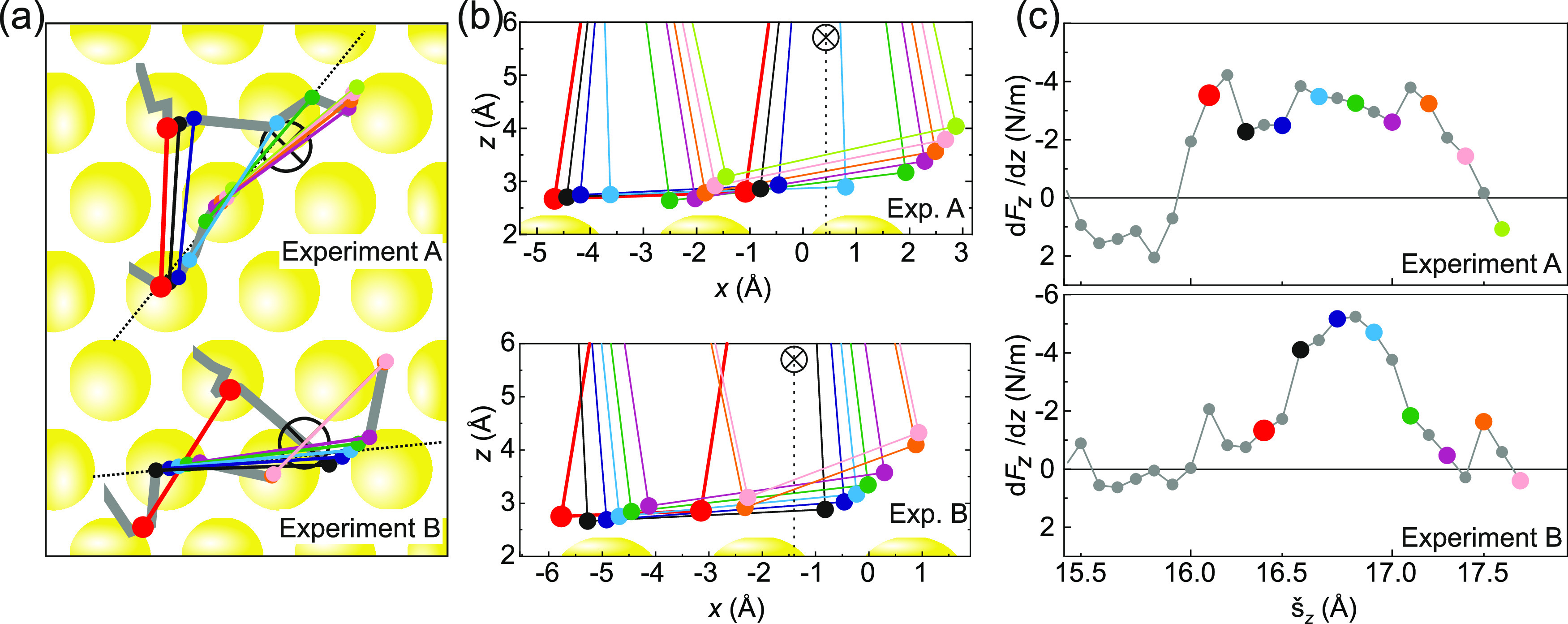
Configuration analysis of vertical molecule. (a) Lateral positions of the two bottom O_carb_ atoms for every second **x̌**_*j*_ in the interval 80 ≤ *j* ≤ 96 (cf. Figures 13g and 14g). The tip position is marked by an encircled cross. The dotted line marks the projection plane used in panel (b). (b) Projection of all PTCDA configurations marked in panel (a) onto a plane that is perpendicular to the surface plane and intersects it along the dotted line in panel (a). PTCDA is abstracted as a quadrangle formed by its four O_carb_ atoms, where only the lower part is shown. The indicated Au atoms are the ones closest to the intersecting planes and likewise projected as is the tip position (dashed line). (c) The best F̌′ curves from Figures 13e and 14e, respectively. Every second F̌_*j*_^′^ value with 80 ≤ *j* ≤ 96 is marked using the color code from panels (a) and (b). Experiment B reaches the limit of the FSA *z*-range already at *j* = 94, such that the last two points are omitted.

With the help of configuration monitoring, we can now chart these differences back to distinctive atomic configurations involving the O_carb_ atoms. To this end, we concentrate on the terminal phase of their traces in Figures 13g and 14g, starting with the last pair of red symbols at *s*_*z*_ = 16 Å. In this phase, the two bottom O_carb_ atoms are detached sequentially from the surface, the last one being the one diagonally opposite the tip. The respective molecular configurations are shown in Figure 15, where they are projected onto the surface plane in panel (a) and onto a plane approximately aligned with the plane of the relevant vertical molecules [dotted line in panel (a)].

In experiment A, the O_carb_ atoms are in energetically favorable positions right on top of Au atoms when being detached. In the first three configurations in Figure 15 (red, black, dark blue), both O_carb_ are bound to Au atoms which are rather far from the tip such that there is already a substantial stress on these bonds even though the molecule is not fully upright yet. Consequently, the main peak starts already at a tip height of *s*_*z*_ = 16 Å. After a jump, the first O_carb_ atom is detached from an on-top position (cyan), and after another jump of the molecular configuration, the second O_carb_ atom is detached from an on-top position as well (violet, orange, rose, light green). This sequence of detachments from strongly bound locations leads to the wide peak of experiment A, as shown in Figure 15c.

For experiment B, the atomic mechanism is different. Here, the red configuration has only one O_carb_ atom in an on-top position. After the first jump, the bottom O_carb_ atom on the side of the tip is detached from a less favorable bridge position (black, dark blue, cyan). At this point, the second O_carb_ atom diagonally opposite of the tip is still on top of a Au atom, but once its detachment starts, it moves away from that position (green, purple). Only at the very end of this process does the lower O_carb_ jump again to the neighboring on-top position (orange, rose), leading to the small spike to the right of the main peak that was discussed above. The peak in experiment B is more intense since the detachment of the first O_carb_ atom happens while the second one is forced to leave its favorable on-top position as well. In experiment A, on the other hand, this detachment happens while the second O_carb_ atom moves into a favorable position.

We note that the two atomic configurations at the bottom end of the molecule in the vertical orientation represent two generic situations that are expected to occur in many lifting experiments. In our view, it is a remarkable success of configuration monitoring that, with nothing else than the two qualitatively different force gradient curves at hand, it has been possible to identify these generic atomic configurations and assign them consistently to either of the two curves.

However, our analysis also illustrates the limits of the configuration monitoring on the basis of force gradient data *F*′(*s*_*z*_). In spite of overall similarity, there clearly is a visible discrepancy between the **x̂**_*j*_ and **x̌**_*j*_ traces for *z* < 14 Å, both in experiments A and B (Figures 13g and 14g). Yet, a comparison of the corresponding F_*j*_^′^ sequences in panel (e) reveals that there is only a very small difference between F̂_*j*_^′^ and F̌_*j*_^′^. We can thus conclude that it would barely be possible for the PF to distinguish between both configuration estimates based solely on the measured *F*′(*s*_*z*_) data in this region. This can in fact be understood as a consequence of the comparatively weak O_carb_–metal interaction on the Au(111) surface. Unlike on the Ag(111) surface, where the oxygen–metal bonds are relatively strong and therefore deform the flat-lying PTCDA molecule by reducing the Ag–O_carb_ distance,^39^ on Au(111) flat-lying PTCDA remains undistorted, with a relatively large oxygen–metal distance. This will reduce the lateral corrugation felt by the O_carb_ atoms, which in turn means a weak dependence of the potential energy landscape on the molecular configuration^48^ at small *s*_*z*_—hence the difficulty of the PF to differentiate between different **x**_*j*_. In contrast, at larger *s*_*z*_, the O_carb_ atoms may approach the Au surface further because the entire molecule–surface vdW attraction is balanced only by the repulsion between the O_carb_ atoms and the surface (Figure 1a). As a consequence, the potential energy landscape becomes more corrugated, and details of molecular configurations have a stronger impact on *F*′, as can be seen when comparing experiments A and B (Figure 1b).

## Conclusions

The future utility of two-contact single-molecule manipulation depends heavily on our capability to monitor molecular configurations. This is true for both fundamental and applied research: known configurations substantially improve our ability to interpret, for example, charge transport experiments through single molecules, and they thoroughly upgrade our control over molecular nanofabrication processes. Here, we presented and benchmarked an approach that turns configuration monitoring into reality. It overcomes the most important challenges which in the past have proved to be showstoppers: the need for accurate yet fast simulations, the disparity between a scalar observation quantity and a high-dimensional unknown molecular configuration, the vast configuration space to be searched, and the need to operate on the few-minutes time scales of typical experiments. Our approach counters these challenges by combining a finite-state automaton (FSA) to store and rapidly access the results of atomistic molecular simulations and a particle filter (PF) to search for likely manipulation trajectories, given an input sequence of observations.

A particular strength of our concept is its generic nature, which allows for flexibility and modularity when applied in a wide range of scenarios: most notably, it works irrespective of the physical observable(s) that is (are) used for configuration monitoring. While the force gradient *F*′ is an obvious choice because it is frequently available in scanning probe microscopy instrumentation, the use of conductance, vibrational spectra, or any other observable with a link to the monitored configurations is also feasible if a corresponding observation model is available. Moreover, since the FSA allows separating the computation of the state transition and observation models from the configuration monitoring itself, the nature of these models is not restricted to a specific choice. While we utilized a tailor-made molecular mechanics simulation in this work, particularly promising future option are machine-learned models trained on high-quality DFT data. Strong research efforts are currently dedicated toward the development of such models; they promise an unmatched combination of accuracy and speed and might ultimately be able to run in real time in a supercomputing facility to react to unexpected events like chemical reactions. Finally, also the particle filter offers a high degree of flexibility. Its performance can be adjusted broadly to different application scenarios by choosing targeted hyperparameters, the most important one being the number of particles. Our specific implementation of the particle filter combines the capability to find good solutions in far-away regions of configuration space with the ability to find a consistent manipulation trajectory. In summary, we have developed and implemented a strategy that firmly establishes molecular configuration monitoring in real time with the limited computational resources typically available in a lab environment.^49^

Based on this advance, we have been able to assign systematic differences in generic features of a common observable (force gradient) to well-defined atomic configurations for the first time in an unbiased statistical evaluation of the experimental data against an exhaustive database of possible structures. This proves the opportunities for configuration monitoring. In the future, such information could provide a much-needed input in the field of molecular electronics, where the interpretation of conductance spectra would benefit enormously from an objective determination of the atomic structure of the molecule–metal interface.

An aspect that we have only briefly touched upon is the automation of molecular manipulation in a robotic manner.^3,50,51^ The concept of a partially observable Markov decision process (POMDP) includes autonomous decision-making in which the policy for selecting an action is based on the agent’s belief regarding the configuration of the molecule, which can be obtained from the particle filter. In the future, configuration monitoring as demonstrated here and decision-making can thus be integrated seamlessly, such that a learning agent takes over the role of the experimenter who, in turn, sets up the rewards which control the agent’s behavior, thereby steering it toward a desired target (manipulation goal). In the past, we have demonstrated this concept in combination with reinforcement learning, albeit operating on very sparse information about the configuration of the molecule.^3^ In synergy with configuration monitoring, such an approach would become much more versatile and efficient.

## Appendix

### Information Gathering

Above, we outline a universal formalism to calculate the average length of a successful information-gathering trajectory. In order to minimize the number of required parameters, we use a model in which the true signal *X*, the additive noise *Z*, and the actually measured value *Y* = *X* + *Z* are continuous, normally distributed random variables with zero mean. Since *X*, *Y*, and *Z* are continuously distributed, the mutual information *I*(*X*, *Y*) has to be determined from the differential entropy *h* of the respective distributions, which is defined for a function *f*(*y*) as^38^

12

Here, *Y* describes a distribution and *y* denotes individual values of the distributed variable. In our specific case where *X* and *Z* are uncorrelated, this mutual information is *I*(*X*, *Y*) = *h*(*Y*) – *h*(*Z*).^38^ The differential entropy of a normal distribution *Y* with standard deviation σ_*Y*_ equals^37^

13

such that the information content of a single measurement becomes

14

Here, σ_*Y*_ and σ_*Z*_ denote the standard deviations of the distribution of (noisy) force gradients *F*′ and of the additive noise *Z*, respectively. Since *Y* is a sum over two uncorrelated normal distributions, σ_*Y*_ can be expressed as , where σ_*X*_ is a parameter obtained from the FSA (Figure 8b) and σ_*Z*_ is a parameter characteristic of the experimental setup. We find a value of σ_*Z*_ = 0.04 N/m in our data by analyzing *F*′(*s*_*z*_) curves with an empty junction.

To incorporate the aspect of correlation into our formalism, we model the two true *F*′ values at neighboring tip positions, choosing *i* = 2 without loss of generality, as two normally distributed random variables *X*_1_ and *X*_2_ with a Pearson correlation coefficient ρ. To compute *I*(*Y*_1_, *X*_2_) in this more complex case, we use the relation^38^

15

where *h*(*X*_2_, *Y*_1_) is the differential entropy of the bivariate normal distribution (*X*_2_, *Y*_1_). This entropy is given by^52^

16

with |Σ| denoting the determinant of the covariance matrix of (*X*_2_, *Y*_1_)

17

The off-diagonal elements of Σ require the calculation of the covariance *cov*(*X*_2_, *Y*_1_), which is not known but can be obtained from known quantities as

18

Here, we have taken advantage of the fact that the true signal *X* and the noise *Z* are uncorrelated, such that ρ_*XZ*_ = 0. The covariance matrix of (*X*_2_, *Y*_1_) is thus

19

Consequently, the expression for the differential entropy (eq 16) is

20

and the corresponding expression for the mutual information content is (eqs 13 and 15)

21

In the limit of ρ = 1, *X*_1_ and *X*_2_ are identical and, consequently, the simpler relation in eq 14 is obtained in this limit. Finally, the expression for the additional information Δ*I*(*X*_2_, *Y*_2_) = *I*(*X*_2_, *Y*_2_) – *I*(*X*_2_, *Y*_1_) gained from observing *Y*_2_ is

22

Having obtained this final result, the number of required steps can be computed as *K* = 1 + (*I*(*C*) – *I*(*X*_1_,*Y*_1_))/Δ*I*(*X*, *Y*) because the first measurement yields the full information *I*(*X*_1_,*Y*_1_), while all subsequent measurements only yield Δ*I*(*X*_*i*_, *Y*_*i*_) = Δ*I*(*X*_2_, *Y*_2_).

## Supporting Information Available

Ad hoc, brute force, and consistent configuration estimates **x̃**, **x̂**, and **x̌** of the PF change as the tip is lifted up (Video S1) (MP4)
Ad hoc, brute force, and consistent configuration estimates **x̃**, **x̂**, and **x̌** of the PF change as the tip is lifted up (Video S2) (MP4)
Detailed analysis of the reversibility of manipulation trajectories, which is relevant for information gathering (PDF)
