# Supplementary material for: Concept for the Real-Time Monitoring of Molecular Configurations during Manipulation with a Scanning Probe Microscope
Source: J Phys Chem C Nanomater Interfaces. 2023 Jul 10;127(28):13817–36. doi: 10.1021/acs.jpcc.3c02072 (PMC10364088; doi:10.1021/acs.jpcc.3c02072)
Supplement: Supplementary file 3 — jp3c02072_si_003.pdf [file jp3c02072_si_003.pdf]

Supporting Information for Concept for the Real-Time Monitoring of Molecular Configurations during Manipulation with a Scanning Probe Microscope

Joshua Scheidt,[†,‡,¶] Alexander Diener,[†,‡,¶] Michael Maiworm,[§] Klaus-Robert Müller,[‖,⊥,#] Rolf Findeisen,[@] Kurt Driessens,[¶] F. Stefan Tautz,[†,‡,△] and Christian Wagner[*,†,‡]

†*Peter Grünberg Institut (PGI-3), Forschungszentrum Jülich, 52425 Jülich, Germany*
‡*Jülich Aachen Research Alliance (JARA)-Fundamentals of Future Information Technology, 52425 Jülich, Germany*
¶*Maastricht University, Data Science and Knowledge Engineering, Maastricht, 6229 EN, The Netherlands*
§*Otto-von-Guericke-Universität Magdeburg, Laboratory for Systems Theory and Automatic Control, 39106 Magdeburg, Germany*
‖*Max Planck Institute for Informatics, 66123 Saarbrücken, Germany*
⊥*Machine Learning Group, Technische Universität Berlin, 10587 Berlin, Germany*
#*Department of Artificial Intelligence, Korea University, Seoul 136-713, South Korea*
@*Control and Cyber-Physical Systems Laboratory, Technische Universität Darmstadt, 64289 Darmstadt, Germany*
△*Experimentalphysik IV A, RWTH Aachen University, 52074 Aachen, Germany*

E-mail: c.wagner@fz-juelich.de

Reversibility of manipulation trajectories

In the supporting information, we analyze reversibility on the level of entire manipulation trajectories, not just on the level of single manipulation steps. Formally, the question whether a given manipulation trajectory is reversible or not can be decided on the basis of the directed graph that underlies the FSA. In principle, in any strongly connected part of that graph (i.e., a subgraph in which every node is reachable from every other node) information gathering with the option of returning to the original state (i.e., the original node of the graph) is possible. However, the return path is generally not known. If the additional criterion of full reversibility at the level of individual manipulation steps was fulfilled everywhere in the subgraph, one possible return path would always be clear, namely the reversed sequence of all visited states.

We now assess the impact of the 2.1% irreversible transitions (most of which are external as shown in Table 1 of the main text) on information gathering trajectories which are designed to explore configuration space before returning the tip to its initial location $\mathbf{s}_0$. For our analysis, we used circular tip trajectories ('rings') that enclose a certain part of $\mathbf{s}$ space (Fig. S1a) as an example. Each of our trial trajectories starts at a randomly chosen initial configuration $\mathbf{x}_0$ and encircles the initial tip position $\mathbf{s}_0$ in either one, two, or three rings in the x, y, and z planes and finally returns to $\mathbf{s}_0$. These rings have radii r ranging from one neighboring FSA state ($r = 0.12\,\text{Å}$) to eight FSA states ($r = 0.96\,\text{Å}$) (Fig. S1b). To obtain a decent statistics, we simulated 3,500 trajectories for each parameter combination, summing to a total of 84,000 trajectories.

We distinguish two scenarios. Either the tip returns to $\mathbf{s}_0$ on the shortest path (Fig. S1a) after completing the information gathering trajectory, or retraces the entire previous exploration path in reverse direction, thus exploiting local reversibility, if present.

If the tip returns to $\mathbf{s}_0$ on the shortest path, we find that 70.5% of the 84,000 simulated trajectories had at least one anchor change. However, in 76.4% out of these, the molecule nevertheless returned to the original configuration $\mathbf{r}_0$ via another anchor change. The probability of ending in a different configuration at the end of the entire trajectory increases from 1% for $r = 0.12\,\text{Å}$ and a single ring to 48% for $r = 0.96\,\text{Å}$ and three rings (Table S1). The size comparison of rings and anchor sets in Fig. S1b clearly shows that there is a large probability for the larger rings to leave the initial anchor set. Since only external transitions have a considerable probability of introducing non-reversibility (Tab. 1 of the main text), large rings should have a higher chance of an irreversible configuration change, as is confirmed by the probabilities summarized in Tab. S1. As expected, irreversibility increases with the used number of rings $1 \leq N \leq 3$ and thus the total length of the trajectory, whereas the plane (x, y, or z) in which a single ring is located has practically no effect (not shown). Specifically, we observe that the chance of irreversibility increases with the length of the information gathering trajectory with an approximate rate of $2.5\,\%/\text{Å}$.

Reversing the entire information gathering trajectory step by step does not improve the probability of returning to $\mathbf{r}_0$. On the contrary, the data in Table S1 show that using such extended return trajectories decreases this probability with a rate of approximately $1\,\%/\text{Å}$ trajectory length. While this is smaller than the corresponding rate for the forward trajectory, it is still an undesired effect. Hence, we can conclude that the presence of only 2.1% irreversible transitions is sufficient to spoil the approach of reversing information gathering

Table S1: Global reversibility. Percentage of trajectories that do not return the molecule to its original configuration $\mathbf{r}_0$ after N rings of radius r (Fig. S1). The percentages in brackets indicate the probabilities of not returning to $\mathbf{r}_0$ if the tip is returned to s_0 by reversing the *entire* trajectory (N rings) step by step.

	Radius r of rings			
N	0.12 Å	0.36 Å	0.60 Å	0.96 Å
1	1.1 (2.1)%	7.5 (11.5)%	14.2 (20.7)%	24.5 (33.6)%
2	2.7 (4.5)%	14.5 (18.2)%	22.7 (31.5)%	37.9 (49.8)%
3	3.3 (5.5)%	15.9 (22.7)%	28.1 (37.9)%	48.3 (58.8)%

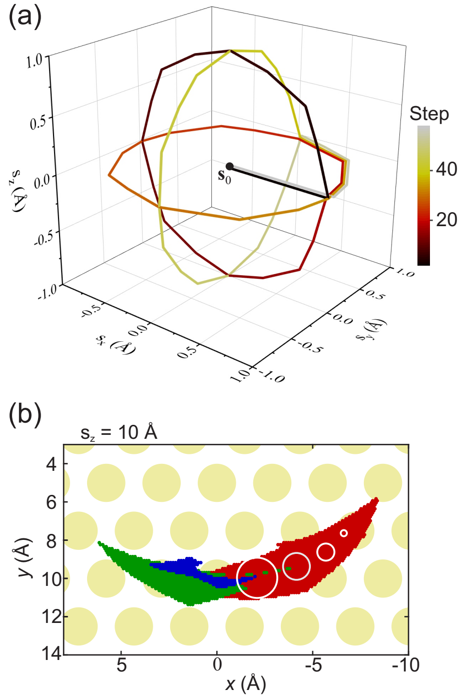

Figure S1: Information-gathering trajectories around a point. **(a)** Circular trajectory created for a radius of 0.96 Å. The trajectory starts at a random tip position (here, $\mathbf{s} = (0, 0, 0)$), moves outwards, follows three circles along the three principal axes and returns to the origin on the shortest possible path (grey line segment). **(b)** Comparison of the four different ring sizes (white, $r = 0.12$ Å to $r = 0.96$ Å) used in our information-gathering trajectories to the lateral extent of the anchor sets at a tip height of $\mathrm{s}_z = 10$ Å (Fig. 6 of the main text). The Au(111) lattice is shown in the background.

trajectories step by step. Instead, such trajectories should be constructed as short as possible, spanning a volume as small as possible and returning to $\mathbf{s}_0$ on the shortest possible path once a sufficient amount of F' data has been acquired. We can put these findings into perspective if we remember that even the largest of the studied circular trajectories has a diameter well below the lattice constant of the Au(111) surface of 2.884 Å (Fig. S1b). Hence, an information gathering trajectory in molecular manipulation experiments should not leave a volume which is, in all dimensions, small compared to the lattice constant of the surface. Of course, this limits the amount of information that can be gathered. In the end, designing an information gathering trajectory will involve a trade-off between information gain and risk of irreversibility.
